# Supplementary material for: Near quantitative synthesis of urea macrocycles enabled by bulky N-substituent
Source: Nat Commun. 2021 Mar 10;12:1572. doi: 10.1038/s41467-021-21678-3 (PMC7947004; doi:10.1038/s41467-021-21678-3)
Supplement: Supplementary file 1 — Supplementary Information [file 41467_2021_21678_MOESM1_ESM.pdf]

Supplementary Information for

Near quantitative synthesis of urea macrocycles enabled by bulky *N*-substituent

Yingfeng Yang[1,‡], Hanze Ying[1,‡], Zhixia Li[2,3], Jiang Wang[4], Yingying Chen[1], Binbin Luo[1], Danielle L. Gray[5], Andrew Ferguson[6], Qian Chen[1,3,7,9], Y Z[2,3], Jianjun Cheng[1,3,7-9]*

[1] Department of Materials Science and Engineering, University of Illinois at Urbana-Champaign, Urbana, Illinois 61801, United States.

[2] Department of Nuclear, Plasma, and Radiological Engineering, University of Illinois at Urbana-Champaign, Urbana, Illinois 61801, United States.

[3] Beckman Institute for Advanced Science and Technology, University of Illinois at Urbana- Champaign, Urbana, Illinois 61801, United States.

[4] Department of Chemistry, Rice University, Houston, Texas 77005, United States

[5] George L. Clark X-Ray Facility & 3M Materials Laboratory, School of Chemical Sciences, Urbana, Illinois 61801, United States.

[6] Institute for Molecular Engineering, Chicago, Illinois 60637, United States.

[7] Department of Chemistry, University of Illinois at Urbana-Champaign, Urbana, Illinois 61801, United States.

[8] Department of Bioengineering, University of Illinois at Urbana-Champaign, Urbana, Illinois 61801, United States.

[9] Materials Research Laboratory, University of Illinois at Urbana-Champaign, Urbana, Illinois 61801, United States.

‡ These authors contributed equally

Corresponding Author * jianjunc@illinois.edu

This PDF file includes:

Supplementary text

Supplementary Figures 1 to S57

Supplementary Tables 1 to 7

Supplementary References

Other supplementary materials for this manuscript include the following:

Single crystals structures (.cif files) S1 to S4

Table of contents

1. Materials and instrumentation 3
   1.1. Materials 3
   1.2. Instrumentation 3
2. Computational Methodology 4
3. Experimental procedures 7
   3.1. Characterization of HUM1 7
      3.1.1. HUM1 characterization 7
      3.1.2. Temperature, concentration and solvent dependence of HUM1 formation 8
   3.2. Synthesis and characterization of a HUM library 10
      3.2.1. Synthesis of A2~A7 10
      3.2.2. Generation of the macrocycle library and calculation of the yields 13
      3.2.3. Structures of the building blocks and macrocycles 14
      3.2.4. Monitoring of the macrocycle formation kinetics 16
      3.2.5. Representative NMR spectra 16
      3.2.6. Self-sorting during the macrocycle formation 31
   3.3. Validation for the role of the *tert*-butyl group 32
      3.3.1. Synthesis of the model compounds 32
      3.3.2. NOE of the model compounds 34
      3.3.3. DFT calculations 36
      3.3.4. Exchange reaction of two macrocycles and their model compounds 39
   3.4. Role of t-Bu group in thermodynamic stabilization 40
      3.4.1. Control experiment 40
      3.4.2. Concentration dependent NMR and NOE studies 41
      3.4.3. Simulation results 44
   3.5 De-*tert*-butylation 46
      3.5.1. Self-assembled fiber of UM1 46
      3.5.2. Binding of UM1 with anions 48
      3.5.3. Synthesis of the De-[N2A4]-C12 and its antimicrobial activity test 49
4. Crystallographic Data 51
5. References 59

1. Materials and instrumentation

1.1. Materials

All reagents were purchased from Sigma-Aldrich or TCI and used as received unless otherwise noted. Deuterated chloroform ($CDCl_3$) was purchase from Cambridge Isotope Laboratory. Tetrahydrofuran (THF) were dried with a column packed with alumina. HPLC grade 0.1% TFA-H2O and acetonitrile were purchased from Fisher Scientific Company LLC (Hanover Park, IL, USA).

1.2. Instrumentation

1.2.1. Nuclear Magnetic Resonance (NMR) Spectroscopy

^{1}H and ^{13}C nuclear magnetic resonance (NMR) spectra were recorded on a Varian U500, VXR500, UI500NB or a Bruker Carver B500 spectrometer, with shifts reported in parts per million downfield from tetramethylsilane and referenced to the residual solvent peak. Nuclear overhauser effect (NOE) spectra were recorded on an Agilent VNS 750NB spectrometer. MestReNova 11.0.1 was used to analyze all spectra.

1.2.2. Fourier-transform infrared spectroscopy (FTIR)

Fourier transform infrared (FT-IR) spectra were performed using a Spectrum 100 spectrometer (Perkin Elmer) on a KBr salt plate.

1.2.3. Electrospray ionization mass spectrometry (ESI-MS)

Electrospray ionization (ESI) mass spectrometry was performed on a Waters Quattro Ultima II. Solvent media was 50% Methanol solution with 0.2% formic acid.

1.2.4. High-performance liquid chromatography (HPLC)

HPLC analysis was conducted by Shimadzu LC system (LC-20AT) connected with PDA detector (SPD-M20A). Phenomenex Kinetex Ph-hexyl column (5 µm, 100 mm × 4.6 mm) was used for analysis. Gradient method was adopted using 0.1% TFA-H_2O and acetonitrile as mobile phase.

1.2.5. Gel Permeation Chromatography (GPC)

Gel permeation chromatography (GPC) was performed in chloroform at a flow rate of 1.0 mL/min on a system equipped with a Model1260 Infinity isocratic pump (Agilent Technology) in series with a 717 Autosampler (Waters) and size exclusion columns (50 Å, 100 Å Phenogel columns, 5 µm, 300 × 7.8 mm, Phenomenex) connected in series. An Optilab rEX refractive index detector (Wyatt Technology) operating at a wavelength of 658 nm were used as detector. Samples were filtered through a 0.45 µm PTFE filter before analysis.

1.2.6. Matrix-assisted laser desorption/ionization time-of-flight mass spectroscopy (MALDI-TOF MS)

MALDI-TOF spectra were obtained on a Bruker Daltonics UltrafleXtreme MALDI TOFTOF equipped with a nitrogen laser of 337 nm. The sample was dissolved in chloroform at a concentration of 10 mg/mL. The cationization agent CF_3COONa was dissolved in THF at a concentration of 10 mg/mL. The matrix used was α-Cyano-4-hydroxycinnamic acid (CHCA, Sigma-Aldrich) and was dissolved in THF at a concentration of 10 mg/mL. Solutions of matrix, sample and cationization agent were mixed in a volume ratio of 4/1/1. The mixed solution was spotted (1µL) on the MALDI sample plate and air-dried. All spectra were recorded in reflectron mode.

1.2.7. SEM

The size and morphology of the samples were characterized using a Hitachi S-4800 SEM operated at 10 kV. The samples were first deposited on a clean Si wafer, dried in ambient conditions and then we conducted a Au sputtering to the samples before SEM characterization.

2. Computational Methodology

Molecular dynamics simulation:

Ring model generation:

All-atom molecular models of 1mer, 2mer, 3mer, chloroform solvent were generated using the Automated Topology Builder (ATB) server (http://atb.uq.edu.au) (1) and modeled using the GROMOS 54A7 force field (2). Atomic partial charges were assigned using semiempirical quantum mechanical calculations conducted using the MOPAC method (3) and all molecules carried zero net charge.

Simulation set up

Molecular dynamics simulations were conducted using the GROMACS 4.6 simulation suite (4). Lennard-Jones interactions were shifted smoothly to zero at 1.4 nm, and interactions between unlike atoms specified by Lorentz−Berthelot combining rules (5). Coulomb interactions were treated by Particle Mesh Ewald (PME) with a real-space cutoff of 1.4 nm and a 0.12 nm reciprocal-space grid spacing (6). Bond lengths were fixed to their equilibrium values using the LINCS algorithm (7). Temperature was maintained at 300 K using a Nosé-Hoover thermostat (8) and pressure at 1.0 bar using an isotropic Parrinello−Rahman barostat (9). Newton's equations of motion were integrated using the leap-frog algorithm with time step of 2 fs (10). System configurations were saved for analysis every 2 ps. Calculations were conducted on NVIDIA Quadro K1200 GPU cards achieving execution speeds of about 30 ns/day. Simulation trajectories were visualized using Visual Molecular Dynamics (VMD) (11).

For single macrocycle simulations, one n-mer was placed in a 4×4×4 nm3 box, for multiple macrocycle simulations, 48 1mers, 24 2mers, 16 3mers, 12 4mers, and 8 6mers each was placed in a 8×8×8 nm3 box, this is to conserve the total monomers to be 48. Boxes were then filled with chloroform molecules and subjected to steepest descent energy minimization until the maximum force on any given atom was less than a threshold of 10 kJ/mol·nm. Atomic velocities were initialized from a Maxwell distribution at 300 K, and the systems were then simulated for 50 ns, the non-bonding energies were computed over the equilibrium portion within the last 20 ns. The non-bonding energies only considered intra and inter macrocycle interactions, and ignore solvent-solvent or solvent-macrocycle interactions.

PMF calculation using umbrella sampling

Umbrella sampling of two macrocycles at different center of mass separations were conducted in a box of size of 8×8×8 nm3. 34 individual umbrella sampling runs on the macrocycle COM distance r were conducted over the range r = (0.2 nm, 3.5 nm) with umbrella windows placed uniformly at 0.1 nm intervals and harmonic restraining potentials with force constants of 1000 kJ/mol·nm2 placed at the center of each window (12). Each umbrella simulation was conducted for 5 ns, and the unbiased potential of mean force (PMF) curve W(z) reconstructed from the biased umbrella simulation trajectories using the Weighted Histogram Analysis Method (WHAM) (13, 14) implemented using g_wham in GROMACS 4.6 (15).

Thermodynamic model

Assume that there are N monomers in the solvent, these monomers could aggregate into clusters of k monomers, and the number of the cluster with size k to be n_k, the system could be characterized by the state vector $\{n_k\}$, which satisfies $\sum_k kn_k = N$. So the probability of observing $\{n_k\}$ is:

$$P(\{n_k\}) = \frac{1}{Q} e^{-\beta u(\{n_k\})} e^{\frac{1}{k_B} S(\{n_k\})}$$

Eq.1

Where $u(\{n_k\})$ is the potential energy of the state $\{n_k\}$, $S(\{n_k\})$ is the entropy of the state $\{n_k\}$, $\beta = 1/k_B$, and $k_B$is the Boltzmann constant, Q is the partition function and is also a constant. In Eq.1, entropy term is:

$$S(\{n_k\}) = \sum_k n_k \, k_B \ln\left(\frac{Ve^{5/2}}{\Lambda_k^3 n_k}\right) + \sum_k n_k \, S_k + S_{solv}$$

Eq.2

Where $V = L^3$ is the volume, Λ_k is the thermal de Broglie wavelength of a k-mer:

$$\Lambda_k^3 = \frac{h}{(2\pi k m_{mon} k_B T)^{1/2}}$$

Eq.3

Where h is the Planck constant, m_{mon} is the mass of a monomer. The first term on the r.h.s of Eq.2 is the translational entropy of the particles of interest, the second term is the configuration entropy of a cluster of k monomers, the last term is the solvent entropy, and is independent of $\{n_k\}$. For simplicity, we treat each cluster rigidly and ignore the second term, and set the solvent entropy to be a constant. The energetic term in Eq.1 can be written as:

$$u(\{n_k\}) = \sum_k n_k u_k + u_{inter} + u_{solv}$$

Eq.4

Where $u_k$is the potential energy of a cluster of k monomers, $u_{inter}$is the energy of the particle-particle and particle-solvent interactions, $u_{solv}$is the potential energy of the solvent, for simplicity, we treat the last two terms in Eq.4 as constant and independent of $\{n_k\}$.

After plugging Eq.2 and Eq.4 into Eq.1, we obtain:

$$P(\{n_k\}) = \frac{1}{Q} e^{-\beta(u_{solv} - TS_{solv})} \left[\prod_k \left(\frac{Ve^{5/2}}{\Lambda_k^3 n_k}\right)^{n_k}\right] e^{-\beta \sum_k (n_k u_k - T n_k S_k)}$$
$$= \frac{1}{Z}\left[\prod_k \left(\frac{Ve^{5/2}}{\Lambda_k^3 n_k}\right)^{n_k}\right] e^{-\beta \sum_k n_k u_k}$$

Eq.5

Where Z is the partition function and absorbs all assumed constants:

$$Z = \sum_{\substack{\{n_k\}\, s.t. \\ \sum_k k n_k = N}} \left[\prod_k \left(\frac{Ve^{5/2}}{\Lambda_k^3 n_k}\right)^{n_k}\right] e^{-\beta \sum_k n_k u_k}$$

Eq.6

Instead of the number of clusters of size k, $\{n_k\}$, we can define the mass fraction of the clusters of size k, $\{f_k\}$ as:

$$f_k = \frac{k n_k}{N}$$

Eq.7

Where $N = \rho V, \rho$ is the density of the particle of interest, which satisfies $\sum_k f_k = 1$. Since ${}^{\Lambda_1}/_{\Lambda_k} = k^{1/2}$, Eq.5 becomes:

$$P(\{f_k\}) = \frac{1}{Z}\left[\prod_k \left(\frac{k^{5/2} e^{5/2}}{\rho \Lambda_1^3 f_k}\right)^{n_k}\right] e^{-\beta \sum_k N f_k u_k}$$

Eq.8

In the thermodynamic limit: $N \to \infty$, when the system reaches equilibrium, $\{f_k\}$ should maximize $P(\{f_k\})$, or minimize the free energy of the state $\{f_k\}$:

$$F(\{f_k\}) \propto -\ln\big(P(\{f_k\})\big) =$$
$$N\left(\beta \sum_k f_k u_k - \sum_k \frac{f_k}{k} \ln\left(\frac{k^{5/2} e^{5/2}}{\rho \Lambda_1^3 f_k}\right)\right) + \ln(Z)$$

Eq.9

The final state $\{f_k\}$ after system reaches equilibrium can be estimated by minimizing the objective function in Eq.9, subject that $\sum_k f_k = 1$. The remaining unknowns in Eq.9 are u_k, the potential energies for different size of aggregates, which could be measured in molecular simulation.

3. Experimental procedures

3.1. Characterization of HUM1

3.1.1. HUM1 characterization

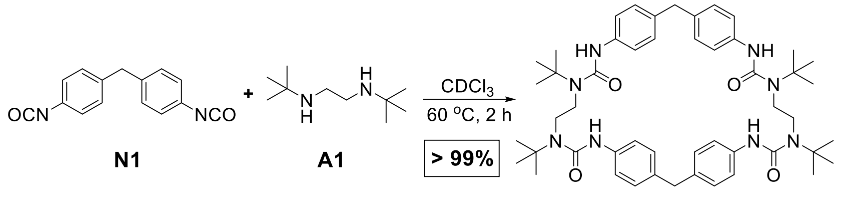

Supplementary Figure 1: Synthesis of **HUM1**

Hindered urea macrocycle 1 (HUM1): Methylene diphenyl diisocyanate (N1, 25 mg, 0.1 mmol) was dispersed in $CDCl_3$ (1 mL) and sonicated. *N,N'*-Di-*tert*-butylethylenediamine (A1, 17.2 mg, 0.1 mmol) in 1 mL $CDCl_3$ was added to the solution of N1. The mixture was capped and incubated at 60 °C for 2 h and then characterized without further purification.

^{1}H NMR (500 MHz, $CDCl_3$): δ 7.93 (s, 4H), 7.42 (d, J = 8.2 Hz, 8H), 7.10 (d, J = 8.2 Hz, 8H), 3.86 (s, 4H), 3.44 (s, 8H), 1.49 (s, 36H). ^{13}C NMR (125 MHz, $CDCl_3$): δ 156.9, 137.6, 135.7, 128.9, 120.2, 55.1, 46.6, 40.8, 30.2. MS-ESI: $C_{50}H_{69}N_8O_4$ [M•H]$^+$, Calculated: 846.1, Found: 846.0.

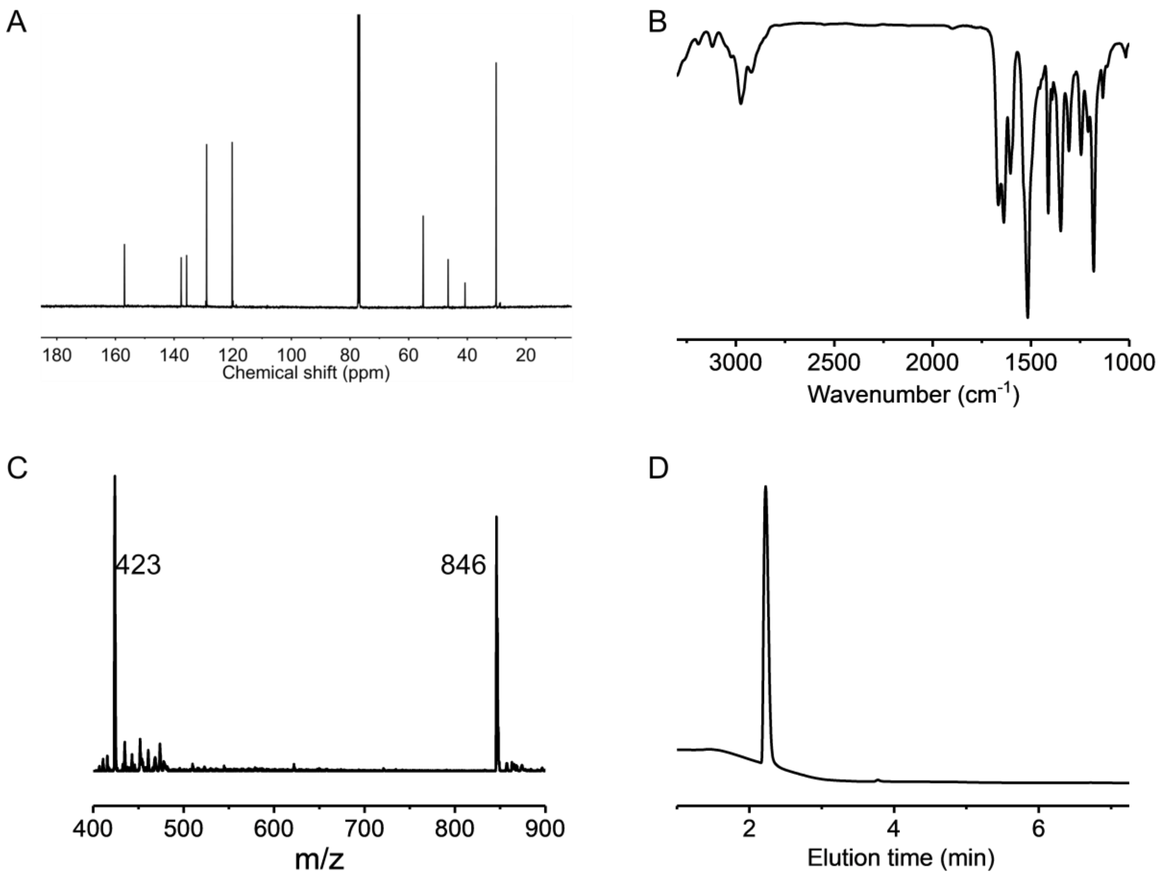

Supplementary Figure 2: Structural characterization of the 1:1 mixture of N1 and A1 after 2 h incubation at 60 °C (50 mM in $CDCl_3$). a ^{13}C NMR spectra (125 MHz); **b** FT-IR spectra; **c** LRMS-ESI spectra; **d** HPLC trace of the mixture.

3.1.2. Temperature, concentration and solvent dependence of HUM1 formation

Temperature: the formation kinetics vary with temperature, but the final yields were all near quantitative from 20 to 75 °C.

N1 and A1 were mixed in 1:1 ratio in $CDCl_3$ (or $C_2D_4Cl_2$ for higher temperature) with a final concentration of 50 mM. The mixture was capped and incubated at 20 °C, 37 °C, 60 °C, 75 °C respectively. Final products and yields were confirmed by ^1H NMR. At higher temperature, HUM1 was formed in near quantitative yield in less than 1 h. At lower temperature, the equilibration took longer because of lower reversibility. At room temperature, the mixture was monitor for 5 days and final yields was also near quantitative. Each group was repeated three times.

Concentration: HUM1 was formed in near quantitative yields with a concentration range from 1 to 500 mM.

N1 and A1 were mixed in 1:1 ratio in $CDCl_3$ with final concentrations of 1 mM, 5 mM, 25 mM, 100mM, 200 mM and 500 mM. The mixtures were capped and incubated at 60 °C. Final products and yields were confirmed by ^1H NMR. All groups gave near quantitative yields after overnight incubation. At higher concentrations, white crystalline precipitates were observed because of lower solubility of the macrocycle. Each group was repeated three times.

Solvent: HUM1 was formed in near quantitative yields in different aprotic solvents.

N1 and A1 were mixed in 1:1 ratio with a final concentration of 50 mM in THF, ethyl acetate, dimethyl formamide and dimethyl sulfoxide. The mixture was capped and incubated at 60 ^{o}C. Final products and yields were confirmed by ^{1}H NMR.

3.2. Synthesis and characterization of a HUM library

3.2.1. Synthesis of A2~A7

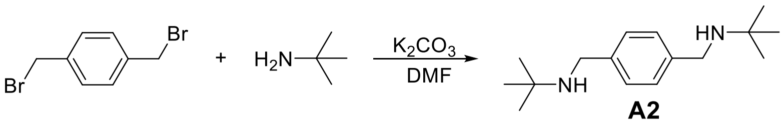

A2

Supplementary Figure 3: Synthesis of compound **A2**

A2: 1,4-Bis(bromomethyl)benzene (264 mg, 1 mmol) was dissolved in 10 mL DMF. *Tert*-butyl amine (438 mg, 6 mmol) and K_2CO_3 (138 mg, 1 mmol) were added to the solution. The suspension was stirred at room temperature for about 24 h and the reaction was monitored by TLC. After completion, the reaction was quenched with 20 mL water, and then extracted with DCM (30 mL × 3). The organic layer was combined, washed twice with brine and then dried with anhydrous Na_2SO_4. Then solvent was removed and crude product was purified by flash column chromatography. Final product was obtained as white powder, yield 95%.

^1H NMR (500 MHz, $CDCl_3$): δ 7.28 (s, 4H), 3.70 (s, 4H), 1.17 (s, 18H). ^{13}C NMR (125 MHz, $CDCl_3$): δ 140.1, 128.4, 50.0, 47.2, 29.3. MS-ESI: $C_{16}H_{29}N_2[M•H]^+$, Calculated: 249.4, Found: 249.6.

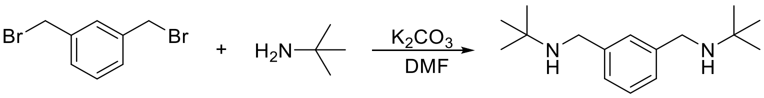

A3

Supplementary Figure 4: Synthesis of compound **A3**

A3: 1,3-Bis(bromomethyl)benzene (264 mg, 1 mmol) was dissolved in 10 mL DMF. *Tert*-butyl amine (438 mg, 6 mmol) and K_2CO_3 (138 mg, 1 mmol) were added to the solution. The suspension was stirred at room temperature for about 24 h and the reaction was monitored by TLC. After completion, the reaction was quenched with 20 mL water, and then extracted with DCM (30 mL × 3). The organic layer was combined, washed twice with brine and then dried with anhydrous Na_2SO_4. Then solvent was removed and crude product was purified by flash column chromatography. Final product was obtained as colorless oil, yield 85%.

^1H NMR (500 MHz, $CDCl_3$): δ 7.32 – 7.22 (m, 2H), 7.23 – 7.17 (m, 2H), 3.71 (s, 4H), 1.18 (s, 18H). ^{13}C NMR (125 MHz, $CDCl_3$): δ 140.1, 128.4, 50.8, 47.2, 29.3. MS-ESI: $C_{16}H_{29}N_2[M•H]^+$, Calculated: 249.4, Found: 249.9.

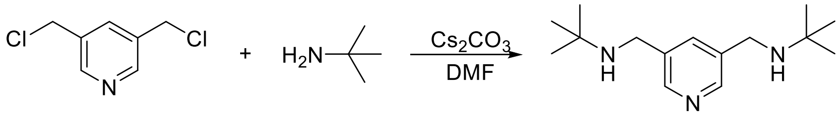

A4

Supplementary Figure 5: Synthesis of compound **A4**

A4: 3,5-Bis(chloromethyl)pyridine (176 mg, 1 mmol) was dissolved in 10 mL DMF. *Tert*-butyl amine (438 mg, 6 mmol) and Cs_2CO_3 (326 mg, 1 mmol) were added to the solution. The suspension was stirred at room temperature for about 12 h and the reaction was monitored by TLC. After completion, the reaction was quenched with 20 mL water, and then extracted with DCM (30 mL × 3). The organic layer was combined, washed twice with brine and then dried with anhydrous Na_2SO_4. Then solvent was removed and crude product was purified by flash column chromatography. Final product was obtained as orange/yellow solid, yield 98%.

^1H NMR (500 MHz, $CDCl_3$): δ 8.43 (s, 2H), 7.68 (s, 1H), 3.72 (s, 4H), 1.18 (d, 18H). ^{13}C NMR (125 MHz, $CDCl_3$): δ 148.3, 136.4, 135.8, 50.9, 44.5, 29.1. MS-ESI: $C_{15}H_{28}N_3[M•H]^+$, Calculated: 250.4, Found: 250.6.

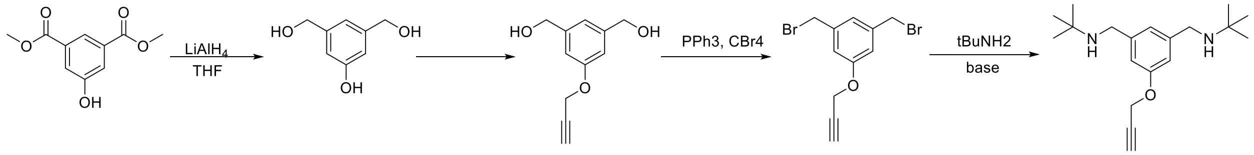

Supplementary Figure 6: Synthesis of compound **A5**

A5: step 1: $LiAlH_4$ (2.277g, 60 mmol) was suspended in 100 mL THF under ice bath. A solution of dimethyl 5-hydroxyisophthalate (4.20 g, 20 mmol, 100 mL THF) was slowly added to the $LiAlH_4$ suspension under vigorous stirring. The mixture was then stirred at 60 ^oC for 2 h and monitored by TLC. After completion, the reaction was quenched by saturated Na_2SO_4 solution (1 mL) and neutralized with concentrated HCl solution (~2 mL). The mixture was then dried with anhydrous Na_2SO_4. The solid was filtered off and the filtrate was concentrated giving (5-hydroxy-1,3-phenylene)dimethanol as colorless oil, which was directly used in the next step. ^1H NMR (500 MHz, $CDCl_3$): δ 6.81 (s, 1H), 6.71 (s, 2H), 4.53 (s, 4H).

Step 2: (5-hydroxy-1,3-phenylene)dimethanol (product from step 1), propargyl bromide solution (2.23 mL, 80 wt. % in toluene, 20 mmol), 18-crown-6 (264 mg, 1 mmol) and K_2CO_3 (2.76 g, 20 mmol) were mixed in 100 mL Acetone and refluxed overnight. After completion, solvent was removed and water (50 mL) was added. The mixture was then extracted with EtOAc (50 mL× 3). The organic layer was combined, washed twice with brine and then dried with anhydrous Na_2SO_4. Then solvent was removed and (5-(prop-2-yn-1-yloxy)-1,3-phenylene)dimethanol was obtained as white solid and directly used in the next step. ^1H NMR (500 MHz, $CDCl_3$): δ 6.97 (s, 1H), 6.88 (s, 2H), 4.69 (d, J = 2.4 Hz, 2H), 4.64 (d, J = 5.1 Hz, 4H), 2.52 (t, J = 2.4 Hz, 1H), 2.17 (d, J = 5.6 Hz, 2H).

Step 3: (5-(prop-2-yn-1-yloxy)-1,3-phenylene)dimethanol (product from step 2) was dissolved in 150 mL anhydrous THF. CBr_4 (9.95 g, 30 mmol) and PPh_3 (7.87 g, 30 mmol) was then slowly added to the solution. The mixture was stirred at room temperature for 4 h. After completion, the reaction was quenched by methanol. Then solvent was removed and crude product was purified by flash column chromatography. 1,3-Bis(bromomethyl)-5-(prop-2-yn-1-yloxy)benzene was obtained as white solid. ^1H NMR (500 MHz, $CDCl_3$): δ 7.05 (s, 1H), 6.94 (s, 2H), 4.71 (d, *J* = 2.3 Hz, 2H), 4.44 (s, 4H), 2.55 (t, *J* = 2.4 Hz, 1H).

Step 4: 1,3-Bis(bromomethyl)-5-(prop-2-yn-1-yloxy)benzene (product from step 3) was dissolved in 100 mL DMF. *Tert*-butyl amine (7.30 g, 100 mmol) and K_2CO_3 (5.52 g, 40 mmol) were added to the solution. The suspension was stirred at room temperature for about 12 h and the reaction was monitored by TLC. After completion, the reaction was quenched with 100 mL water, and then extracted with DCM (100 mL × 3). The organic layer was combined, washed twice with brine and then dried with anhydrous Na_2SO_4. Then solvent was removed and crude product was purified by flash column chromatography. Final product was obtained as colorless oil. Yield 85% over four steps.

^{1}H NMR (500 MHz, $CDCl_3$): δ 6.94 (s, 1H), 6.83 (s, 2H), 4.69 (d, J = 2.4 Hz, 2H), 3.69 (s, 4H), 2.50 (t, J = 2.4 Hz, 1H), 1.16 (s, 18H). ^{13}C NMR (125 MHz, $CDCl_3$): δ 157.8, 143.2, 121.3, 113.1, 78.8, 75.2, 55.8, 50.7, 47.2, 29.1. MS-ESI: $C_{19}H_{31}N_2[M•H]^+$, Calculated: 303.5, Found: 303.7.

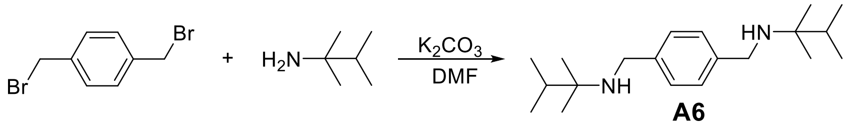

A6

Supplementary Figure 7: Synthesis of compound **A6**

A6: 1,4-Bis(bromomethyl)benzene (264 mg, 1 mmol) was dissolved in 10 mL DMF. 2,3-dimethylbutan-2-amine (506 mg, 5 mmol) and K_2CO_3 (138 mg, 1 mmol) were added to the solution. The suspension was stirred at room temperature for about 24 h and the reaction was monitored by TLC. After completion, the reaction was quenched with 20 mL water, and then extracted with DCM (30 mL × 3). The organic layer was combined, washed twice with brine and then dried with anhydrous Na_2SO_4. Then solvent was removed and crude product was purified by flash column chromatography. Final product was obtained as colorless oil, yield 95%.

^{1}H NMR (500 MHz, $CDCl_3$): δ 7.29 (s, 4H), 3.66 (s, 4H), 1.81 (h, J = 6.8 Hz, 2H), 1.06 (s, 12H), 0.91 (d, J = 6.8 Hz, 12H).

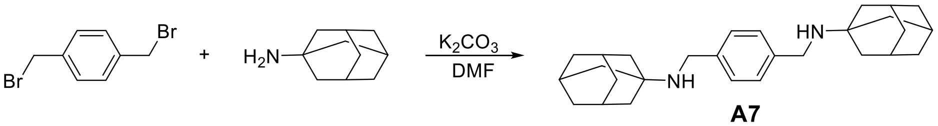

A7

Supplementary Figure 8: Synthesis of compound **A7**

A7: 1,4-Bis(bromomethyl)benzene (264 mg, 1 mmol) was dissolved in 10 mL DMF. Adamantyl amineamine (907.5 mg, 6 mmol) and K_2CO_3 (138 mg, 1 mmol) were added to the solution. The suspension was stirred at 50 ^{o}C for about 24 h and the reaction was monitored by TLC. After completion, the reaction was quenched with 20 mL water, and then extracted with DCM (30 mL*3). The organic layer was combined, washed twice with brine and then dried with anhydrous Na_2SO_4. Then solvent was removed and crude product was purified by flash column chromatography. Final product was obtained as colorless oil, yield 85%.

^{1}H NMR (500 MHz, $CDCl_3$): 7.27 (s, 4H), 3.73 (s, 4H), 2.09 (s, 3H), 1.70 (d, J = 2.9 Hz, 6H), 1.69 – 1.57 (m, 6H).

3.2.2. Generation of the macrocycle library and calculation of the yields

General procedure: The diisocyanate (0.1 mmol) and diamine (0.1 mmol) were mixed directly in $CDCl_3$ (2 mL) and then incubated at 60 °C. The macrocycle formation process was monitored by ^{1}H NMR and further confirmed by ^{13}C NMR and MALDI-TOF. The yields were calculated by the integration from ^{1}H NMR spectra, as is shown by the example below. The NH proton from all species (macrocycles and oligomers) fell into the specified region (6.15~6.4 ppm). The integration of this region was set as 1. The peak at 6.2 ppm came from the target macrocycle and its integration was 0.61. The yield of the macrocycle was considered 0.61 in this case. Different peaks may be chosen to calculate the yields based on how well the peaks can be differentiated.

It should be noted that the macrocycle formation was thermodynamically controlled; thus, factors such as concentration and temperature can affect the final equilibration. Also to achieve high yields of the macrocycles, the exact molar ratio of the building blocks was very important. So the purity of the building blocks and the weighing can affect a lot. For the yields reported here, all the experiments were performed under the above mentioned condition and were repeated three times.

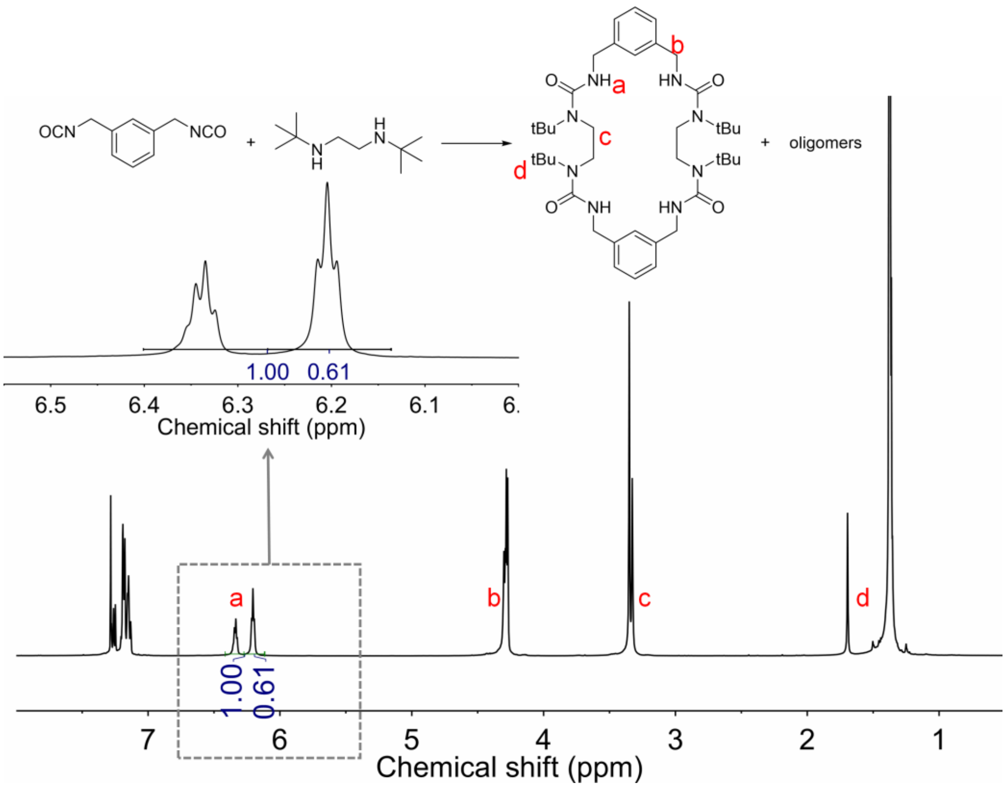

Supplementary Figure 9: Example showing how the yield was calculated.

3.2.3. Structures of the building blocks and macrocycles

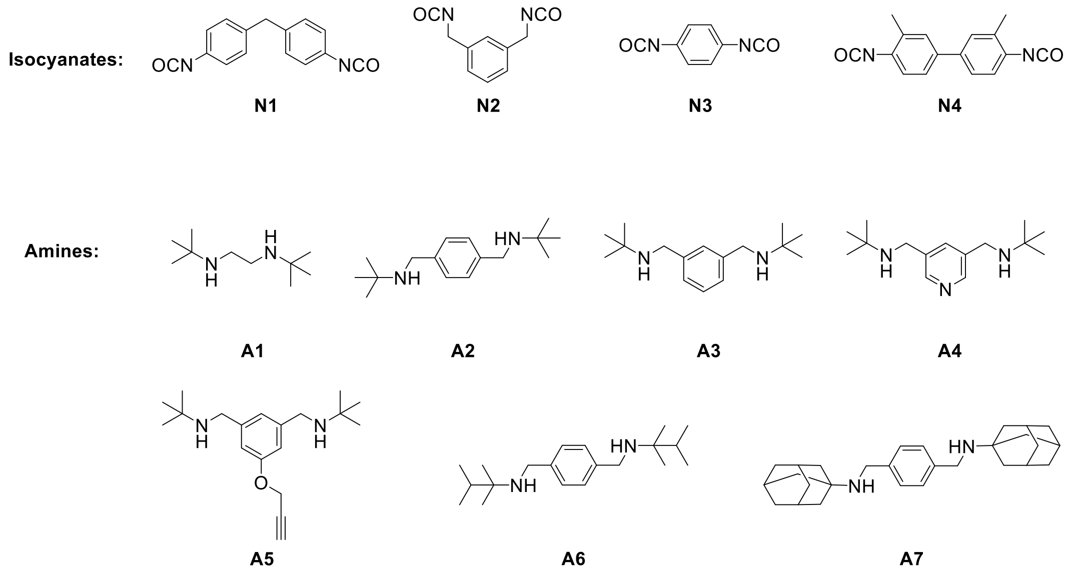

Supplementary Figure 10: Structures of the diisocyanates (N1~N4) and diamines(A1~A5) used in the library.

Structures and yields of the macrocycles were listed below. $[NiAj]_x$ refers to the macrocyle obtained from the combination of the diisocyanate Ni and the diamine Aj, x= 1 or 2. The number refers to its yield under the reaction condition. Since only a few single crystals structures were obtained, the conformations drawn here may or may not be right.

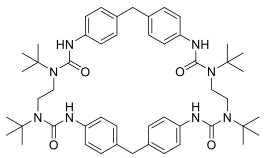

[N1A1]$_2$, >98%

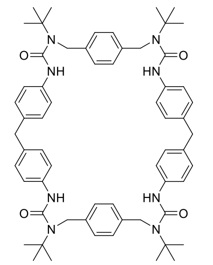

[N1A2]$_2$, 78%

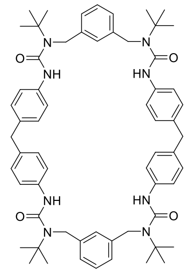

[N1A3]$_2$, 73%

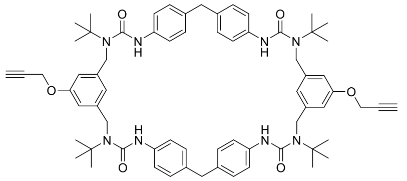

[N1A5]$_2$, 78%

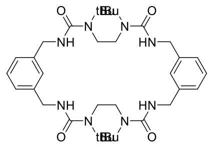

[N2A1]$_2$, 61%

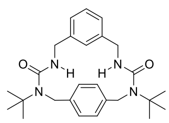

[N1A2]$_1$, >95%

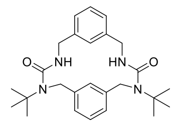

[N2A3]$_1$, >95%

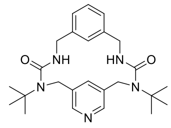

[N2A4]$_1$, >95%

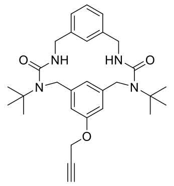

[N2A5]$_1$, 91%

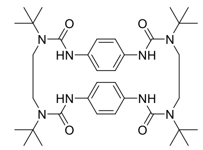

[N3A1]$_2$, 81%

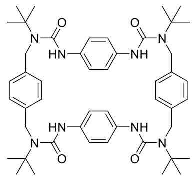

[N3A2]$_2$, >98%

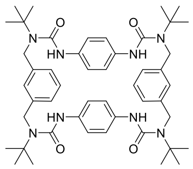

[N3A3]$_2$, >95%

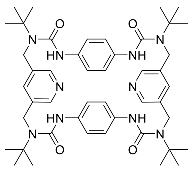

[N3A4]$_2$, >95%

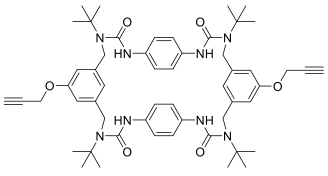

[N3A5]$_2$, >95%

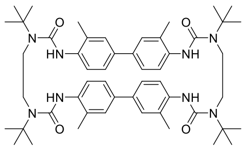

[N4A1]$_2$, 68%

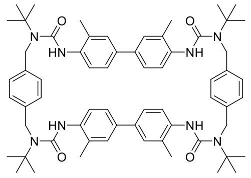

[N4A2]$_2$, >98%

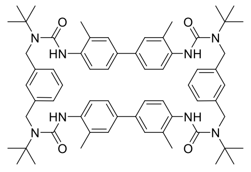

[N4A3]$_2$, >95%

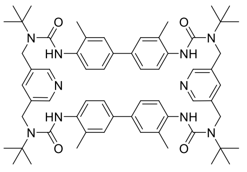

[N4A4]$_2$, >95%

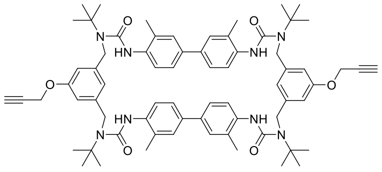

[N4A5]$_2$, >95%

Supplementary Figure 11: Structures of the macrocycles in the library and their yields under experimental condition. [NiAj]$_x$ refers to the macrocycle from the combination Ni and Aj.

3.2.4. Monitoring of the macrocycle formation kinetics

The formation kinetics of some of the combinations (N1~N4 and A1~A3) were tested and monitored by NMR. The diisocyanate (0.1 mmol in 1 mL $CDCl_3$) and diamine (0.1 mmol in 0.5 mL $CDCl_3$) were quickly mixed, rinsed with 0.5 mL $CDCl_3$ and then subjected to NMR immediately. Then the mixtures were kept at 60 ^oC and NMR spectra were taken at various intervals. The yields were calculated by the integration from ^1H NMR spectra, as is shown in 3.2.2.

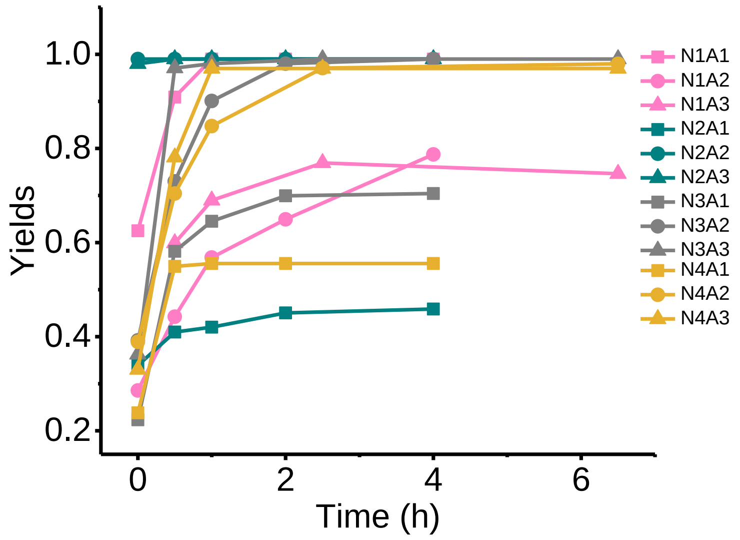

Supplementary Figure 12: Macrocycle formation kinetics of the of different combinations (50 mM in $CDCl_3$, 60 ^oC).

3.2.5. Representative NMR spectra

Several representative ^1H and ^{13}C NMR spectra as well as their corresponding MALDI-TOF spectra were shown below. It should be noted that all the spectra came directly from the mixture **without any purification** (50 mM in $CDCl_3$, incubated at 60 ^oC).

Example 1: $[N2A2]_1$

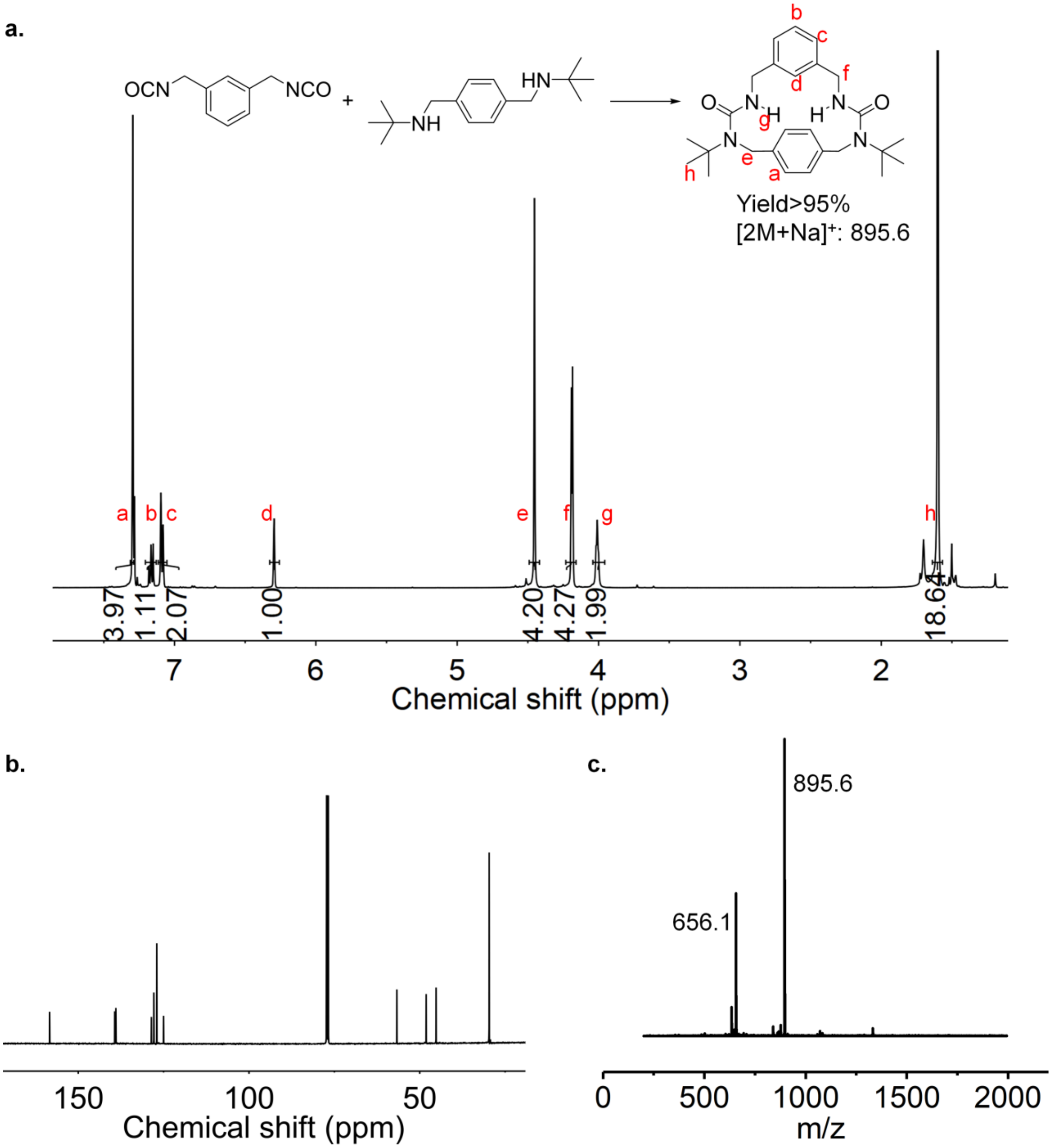

Supplementary Figure 13: a ^{1}H NMR (500 MHz, $CDCl_3$) of $[N2A2]_1$. **b** ^{13}C NMR (125 MHz, $CDCl_3$) of $[N2A2]_1$. **c** MALDI-TOF spectra of $[N2A2]_1$. The peak 656.1 came from the matrix.

Example 2: $[N2A3]_1$

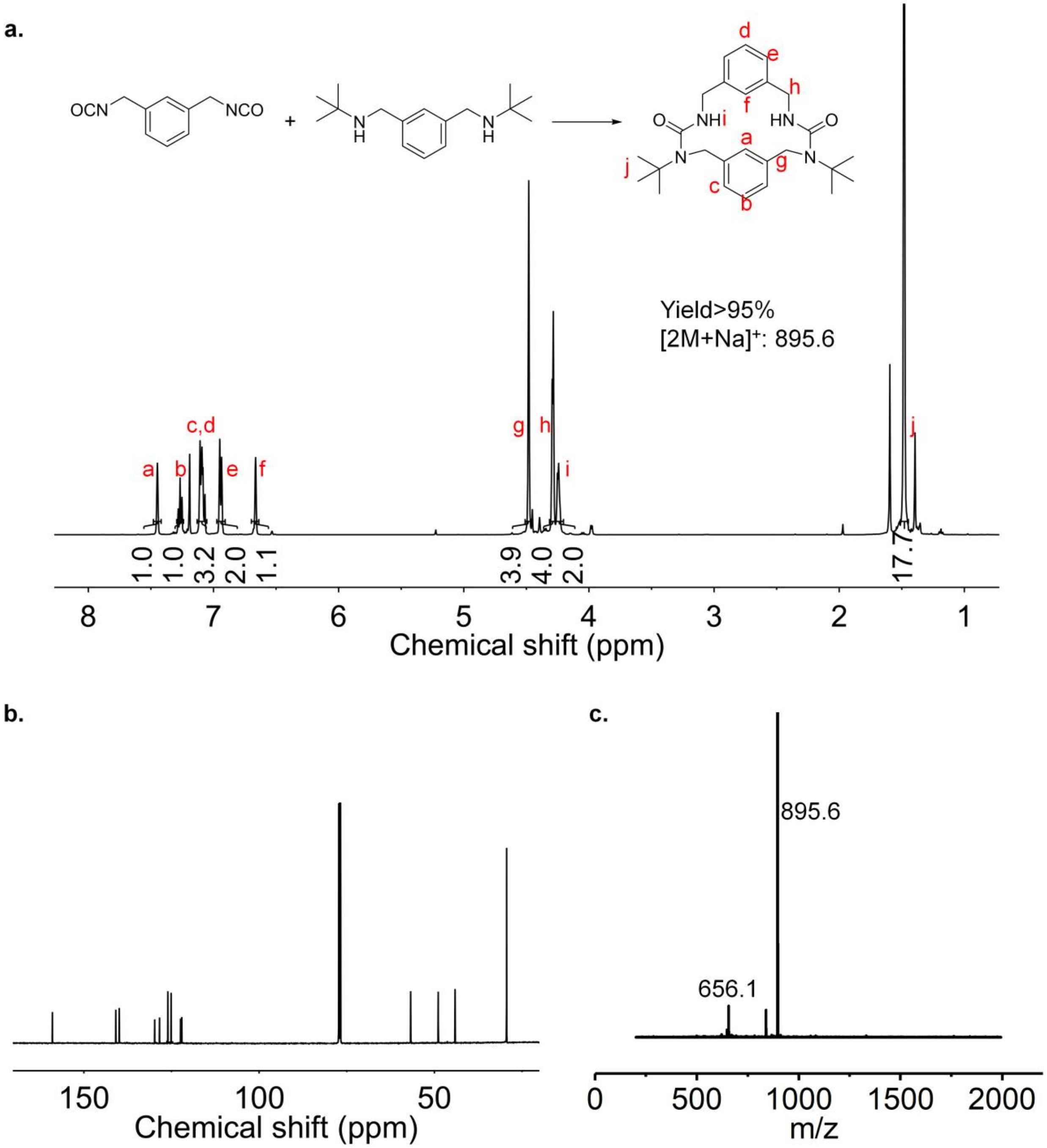

Supplementary Figure 14: a ^1H NMR (500 MHz, $CDCl_3$) of $[N2A3]_1$. **b** ^{13}C NMR (125 MHz, $CDCl_3$) of $[N2A3]_1$. **c** MALDI-TOF spectra of $[N2A3]_1$. The peak 656.1 came from the matrix.

Example 3: [N2A4]₁

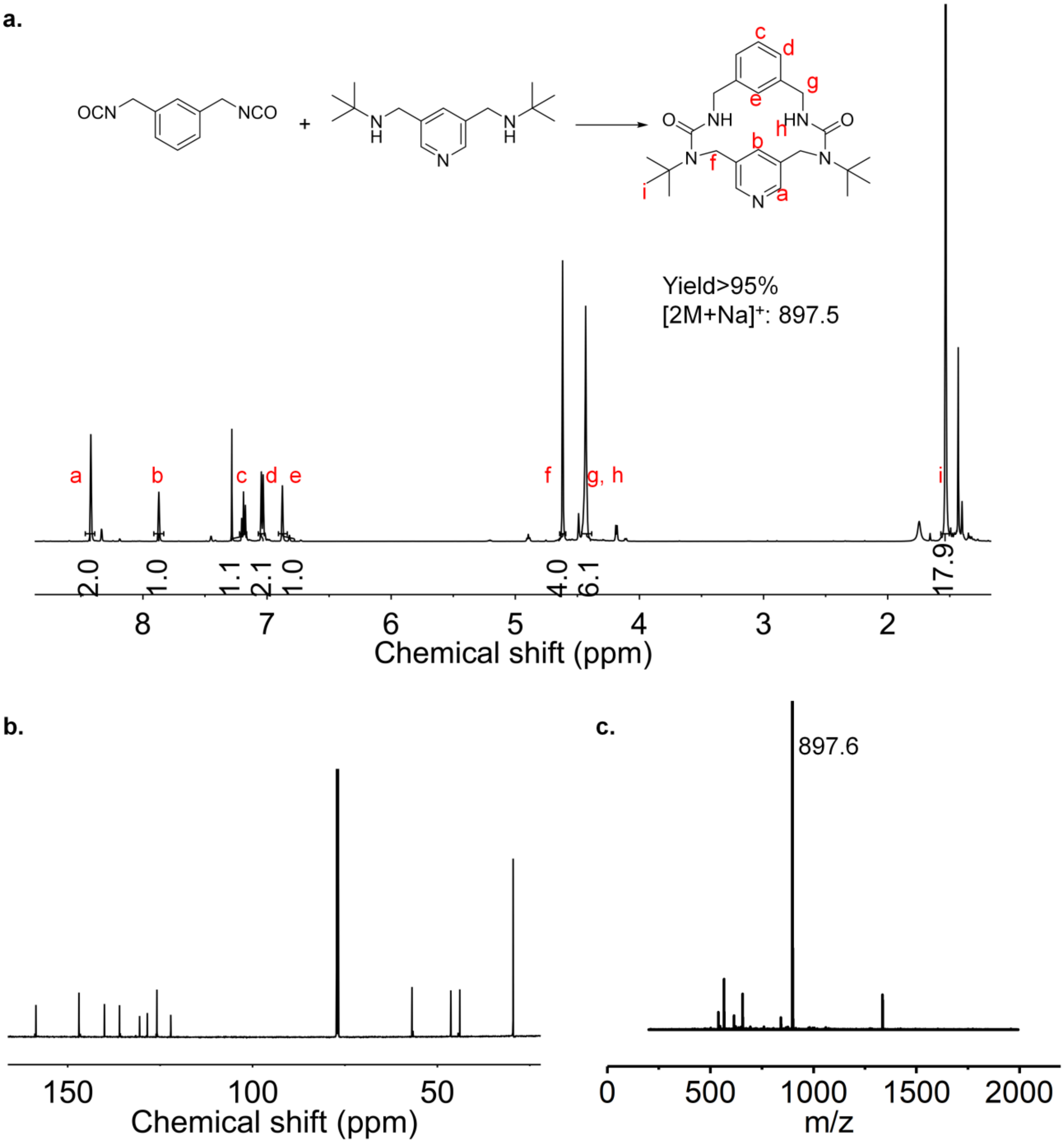

Supplementary Figure 15: a ^{1}H NMR (500 MHz, $CDCl_3$) of $[N2A4]_1$. **b** ^{13}C NMR (125 MHz, $CDCl_3$) of $[N2A4]_1$. **c** MALDI-TOF spectra of $[N2A4]_1$.

Example 4: $[N2A5]_1$

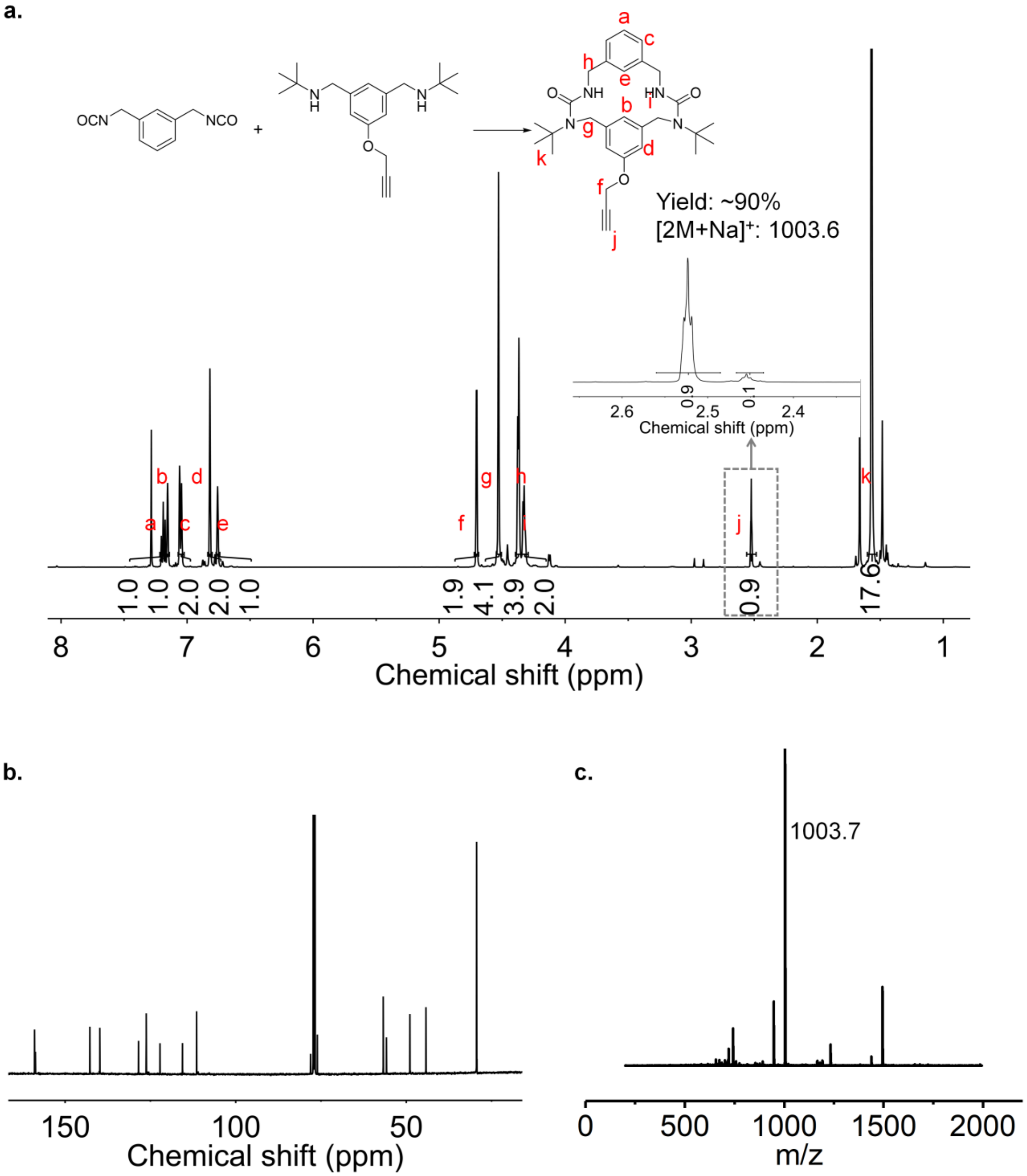

Supplementary Figure 16: a ^{1}H NMR (500 MHz, $CDCl_3$) of $[N2A5]_1$. **b** ^{13}C NMR (125 MHz, $CDCl_3$) of $[N2A5]_1$. **c** MALDI-TOF spectra of $[N2A5]_1$.

Example 5: [N3A2]$_2$

a.

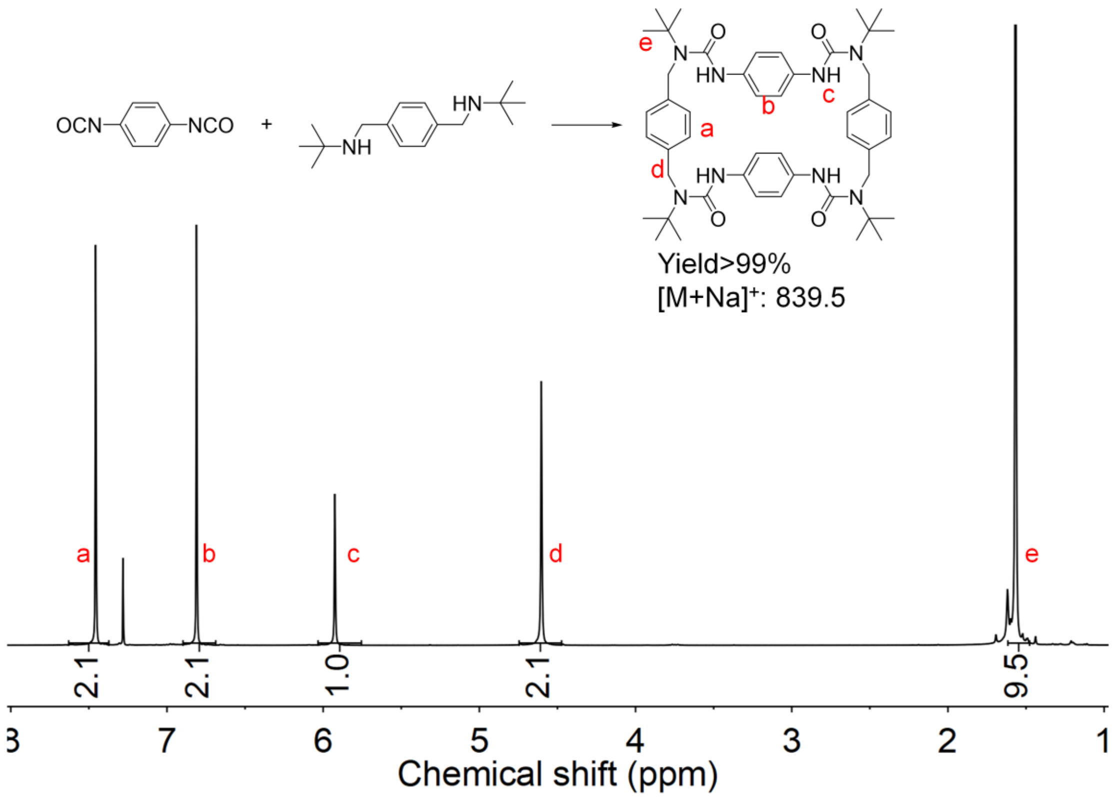

b.

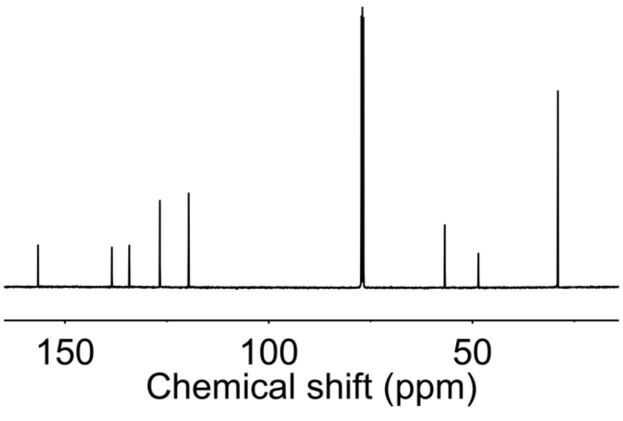

c.

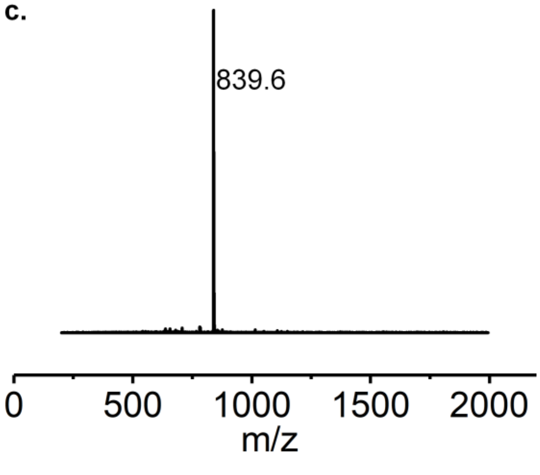

Supplementary Figure 17: a ^{1}H NMR (500 MHz, $CDCl_3$) of [N3A2]$_2$. **b** ^{13}C NMR (125 MHz, $CDCl_3$) of [N3A2]$_2$. **c** MALDI-TOF spectra of [N3A2]$_2$.

Example 6: $[N3A3]_2$

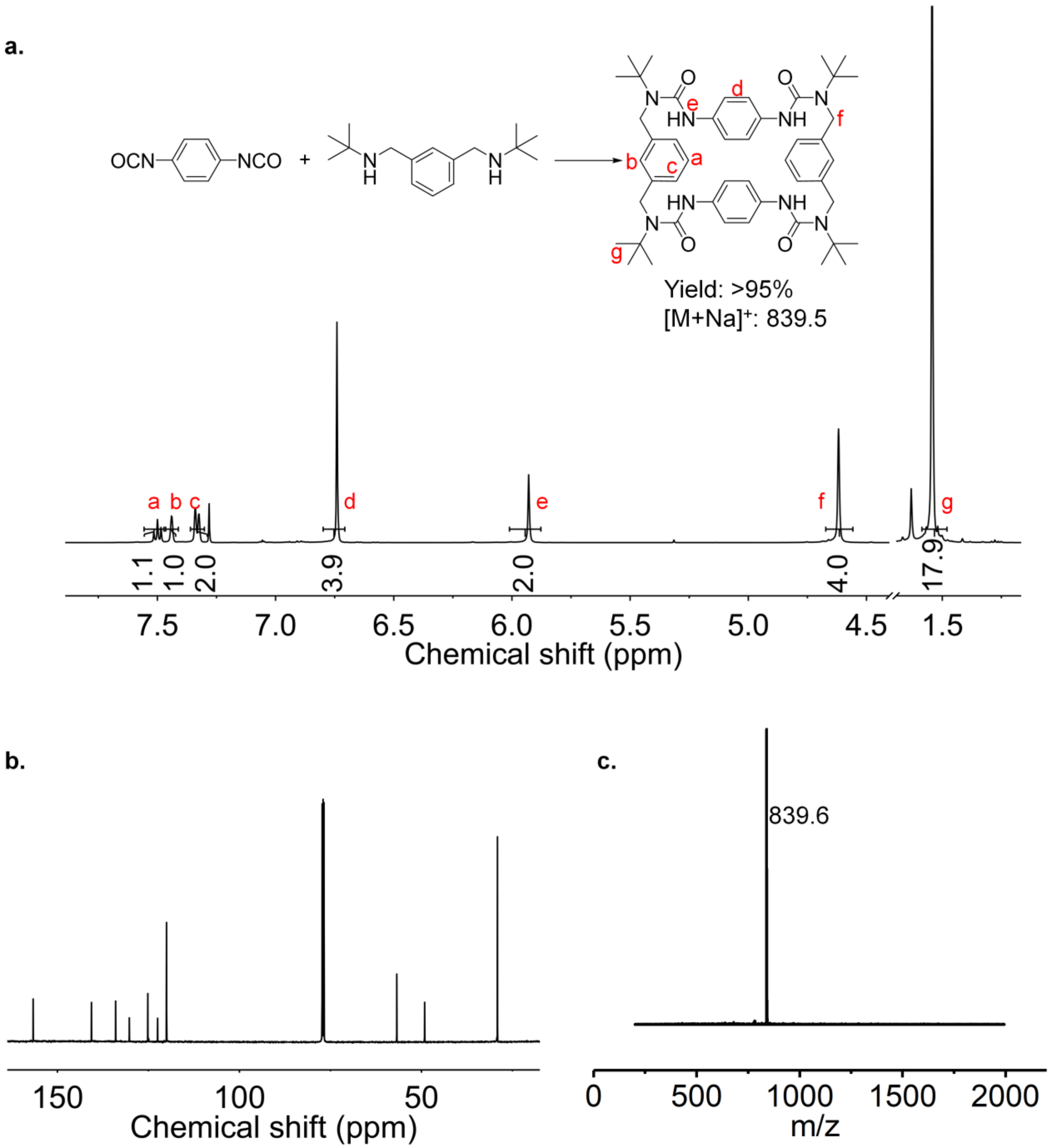

Supplementary Figure 18: a ^{1}H NMR (500 MHz, $CDCl_3$) of $[N3A3]_2$. **b** ^{13}C NMR (125 MHz, $CDCl_3$) of $[N3A3]_2$. **c** MALDI-TOF spectra of $[N3A3]_2$.

Example 7: $[N3A5]_2$

a.

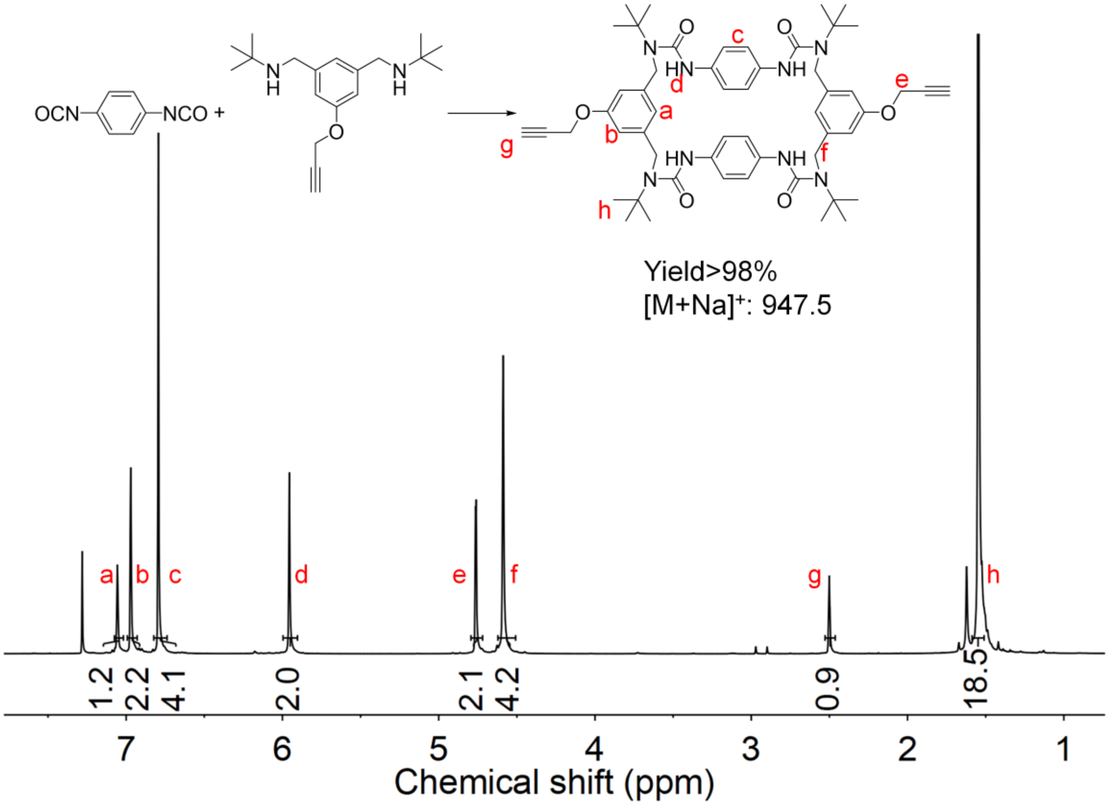

b.

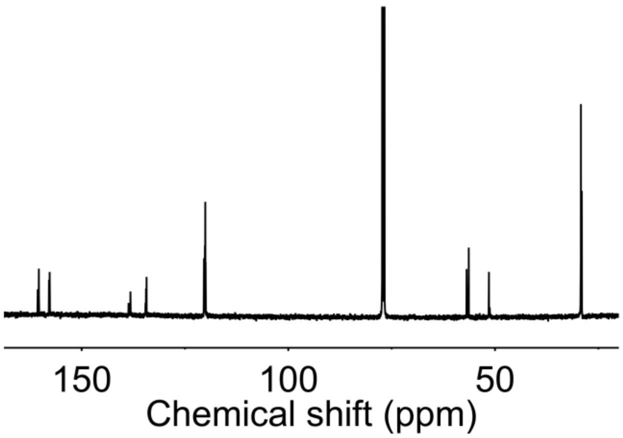

c.

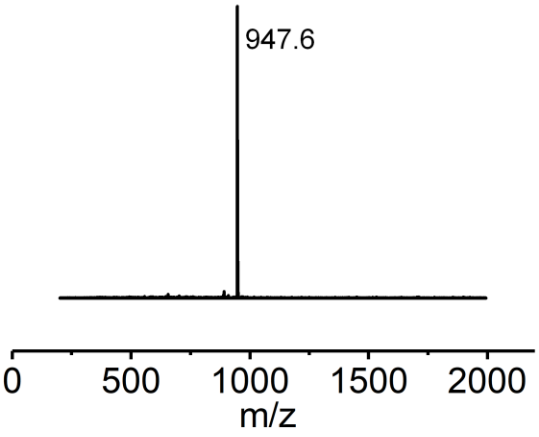

Supplementary Figure 19: a ^{1}H NMR (500 MHz, $CDCl_3$) of $[N3A5]_2$. **b** ^{13}C NMR (125 MHz, $CDCl_3$) of $[N3A5]_2$. **c** MALDI-TOF spectra of $[N3A5]_2$.

Example 8: [N4A2]$_2$

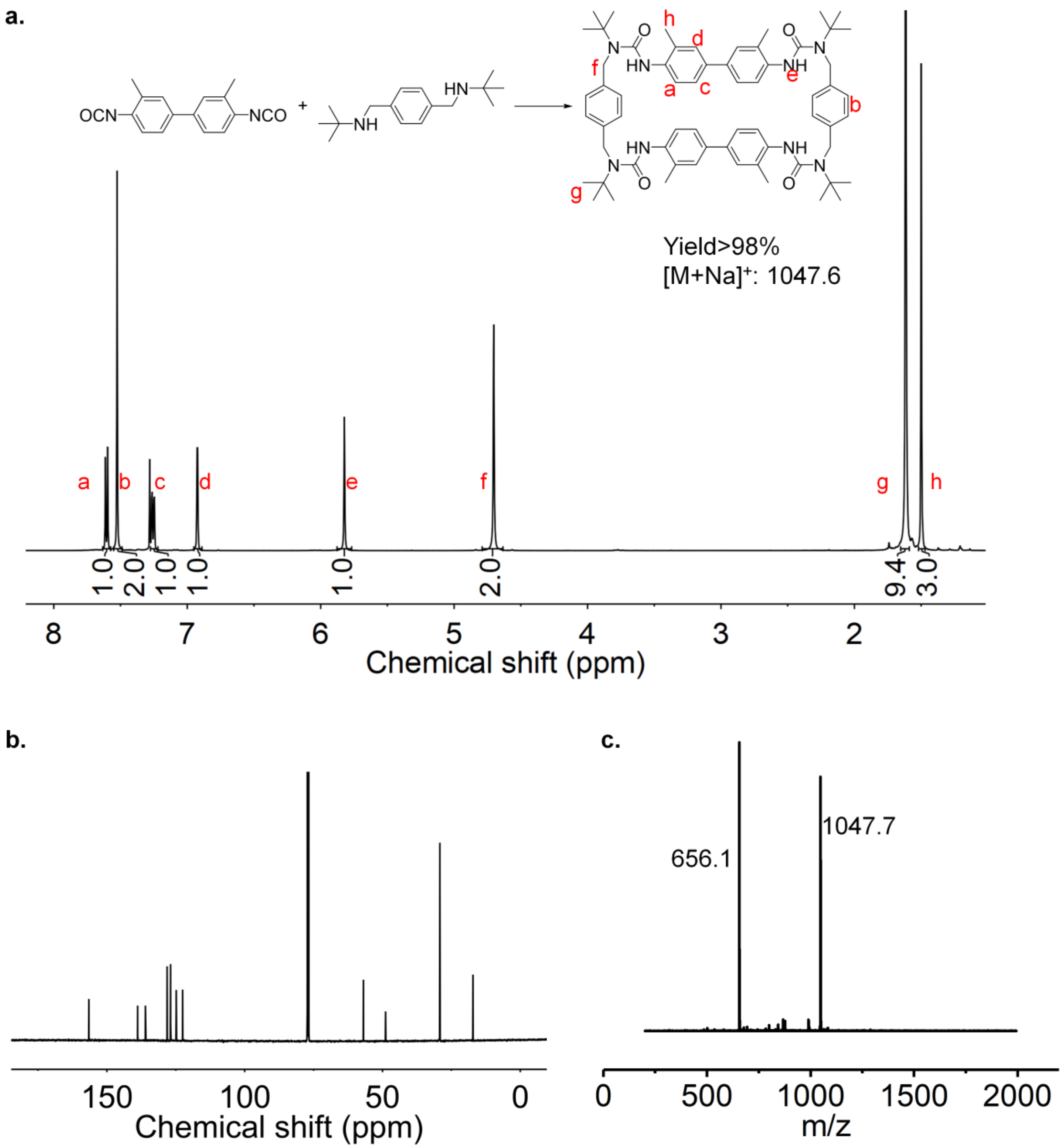

Supplementary Figure 20: a ^{1}H NMR (500 MHz, $CDCl_3$) of [N4A2]$_2$. **b** ^{13}C NMR (125 MHz, $CDCl_3$) of [N4A2]$_2$. **c** MALDI-TOF spectra of [N4A2]$_2$. The peak 656.1 came from the matrix.

Example 9: [N4A3]$_2$

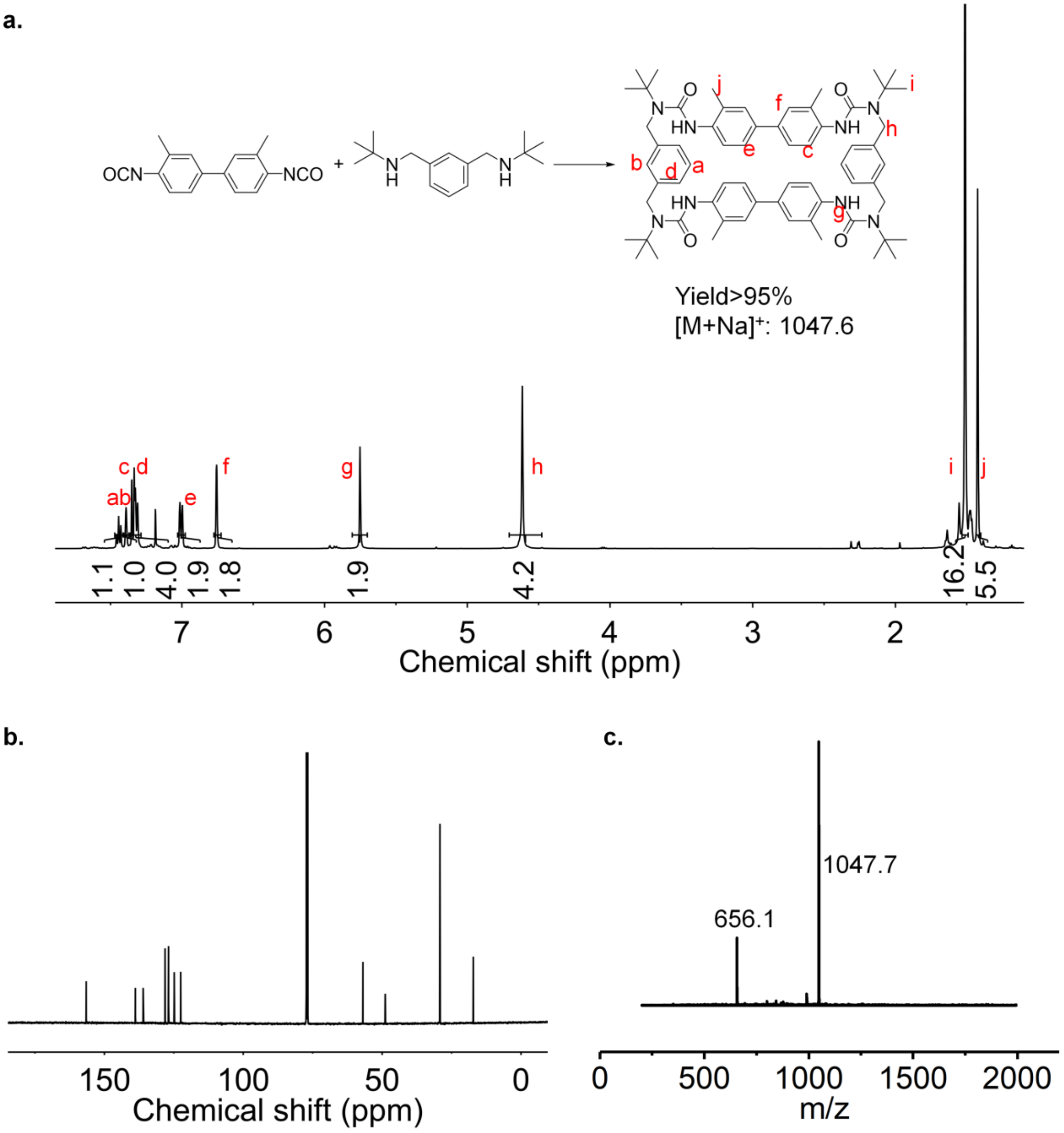

Supplementary Figure 21: a ^{1}H NMR (500 MHz, $CDCl_3$) of [N4A3]$_2$. **b** ^{13}C NMR (125 MHz, $CDCl_3$) of [N4A3]$_2$. **c** MALDI-TOF spectra of [N4A3]$_2$. The peak 656.1 came from the matrix.

Example 10: [N4A5]$_2$

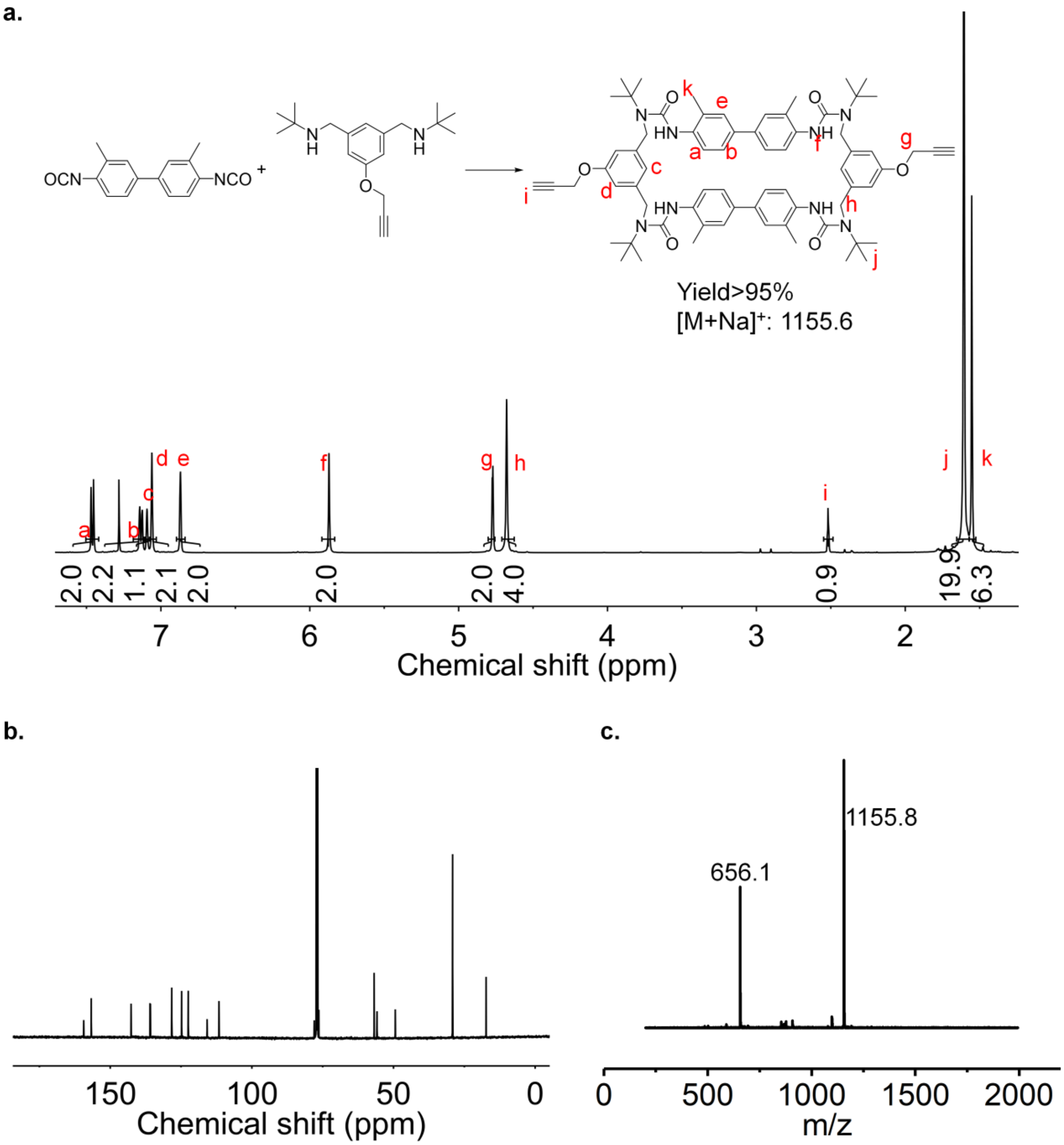

Supplementary Figure 22: a ^{1}H NMR (500 MHz, $CDCl_3$) of $[N4A3]_2$. **b** ^{13}C NMR (125 MHz, $CDCl_3$) of $[N4A3]_2$. **c** MALDI-TOF spectra of $[N4A3]_2$. The peak 656.1 came from the matrix.

Example 11: $[N1A2]_2$

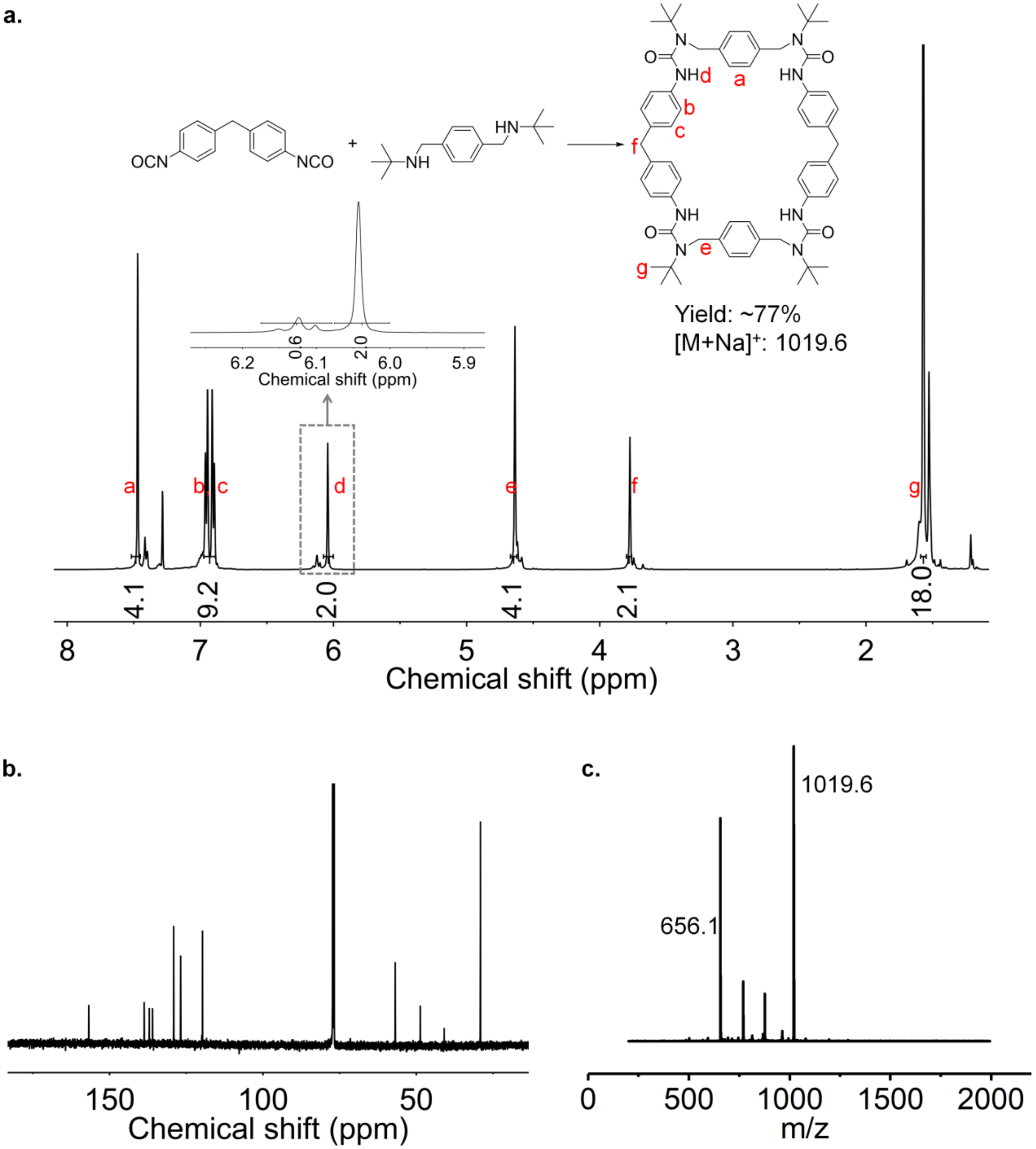

Supplementary Figure 23: a ^{1}H NMR (500 MHz, $CDCl_3$) of $[N1A2]_2$. **b** ^{13}C NMR (125 MHz, $CDCl_3$) of $[N1A2]_2$. **c** MALDI-TOF spectra of $[N1A2]_2$. The peak 656.1 came from the matrix.

Example 12: [N1A3]$_2$

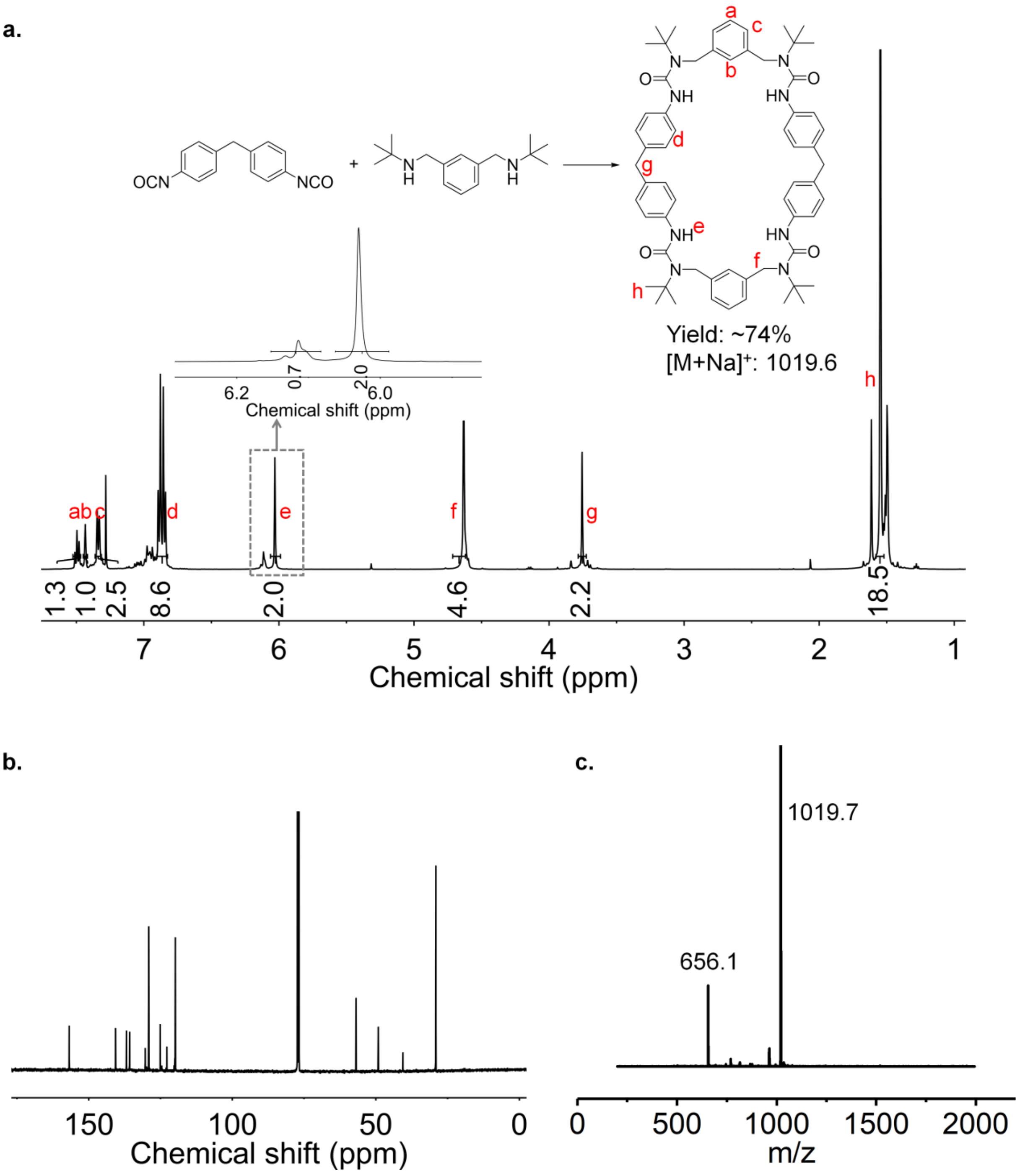

Supplementary Figure 24: a ^{1}H NMR (500 MHz, $CDCl_3$) of [N1A3]$_2$. **b** ^{13}C NMR (125 MHz, $CDCl_3$) of [N1A3]$_2$. **c** MALDI-TOF spectra of [N1A3]$_2$. The peak 656.1 came from the matrix.

Example 13: $[N1A5]_2$

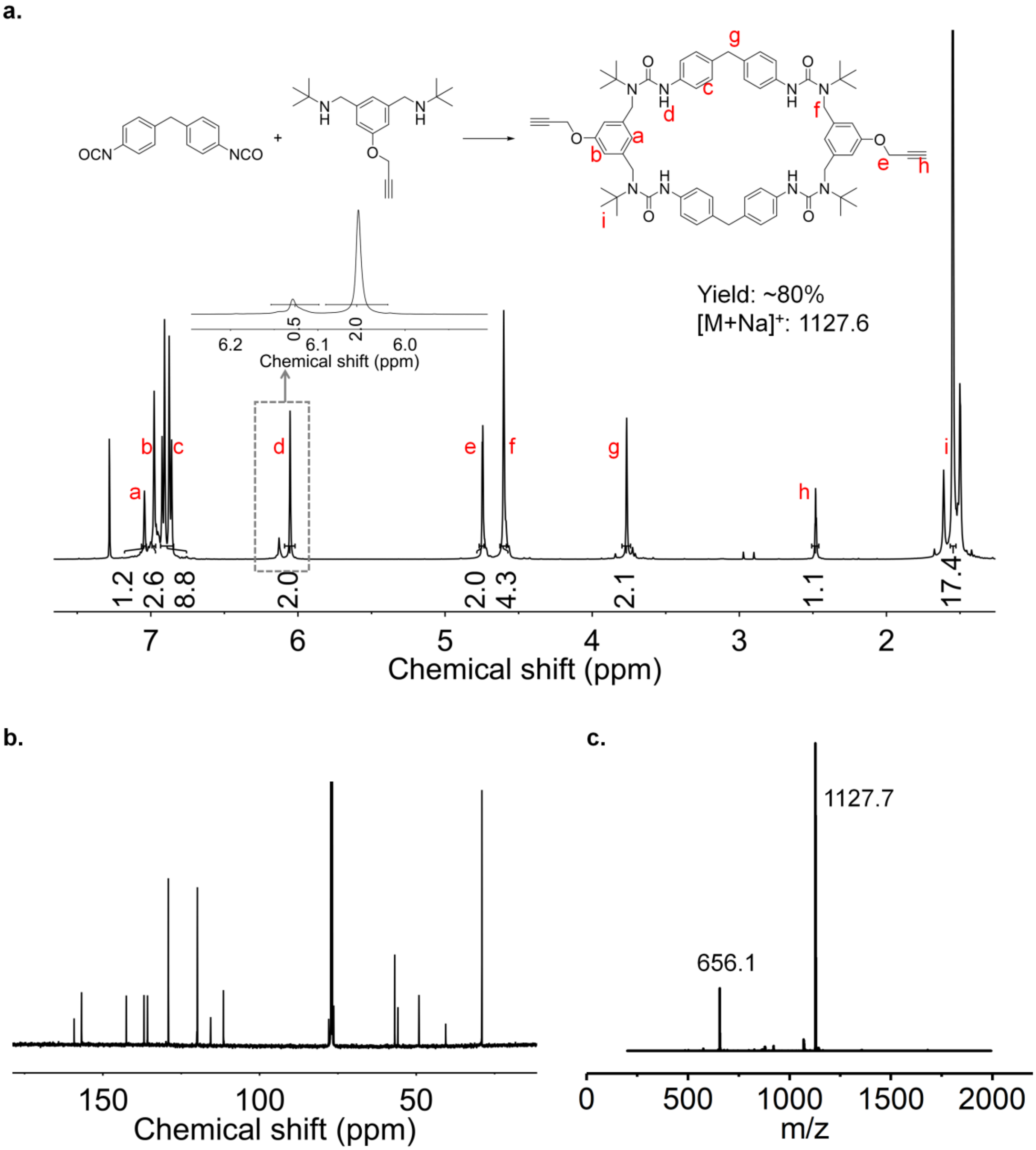

Supplementary Figure 25: a ^{1}H NMR (500 MHz, $CDCl_3$) of $[N1A5]_2$. **b** ^{13}C NMR (125 MHz, $CDCl_3$) of $[N1A5]_2$. **c** MALDI-TOF spectra of $[N1A5]_2$. The peak 656.1 came from the matrix.

[N3A6]$_2$

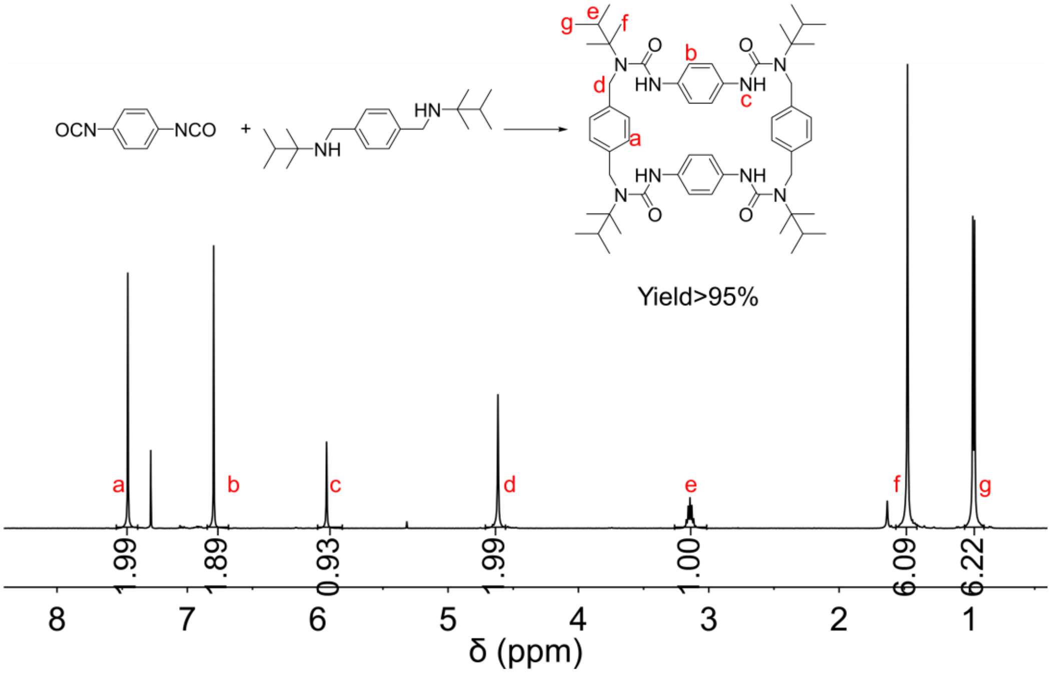

Supplementary Figure 26: ^{1}H NMR (500 MHz, $CDCl_3$) of [N3A6]$_2$.

[N3A7]$_2$

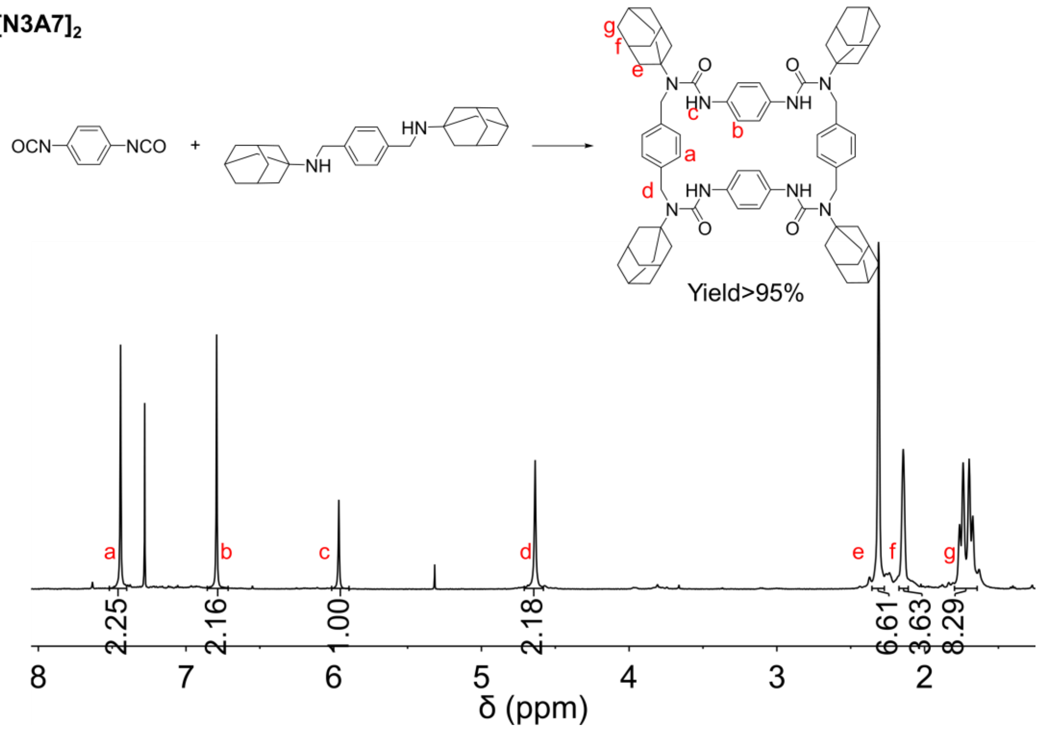

Supplementary Figure 27: ^{1}H NMR (500 MHz, $CDCl_3$) of [N3A6]$_2$.

3.2.6. Self-sorting during the macrocycle formation

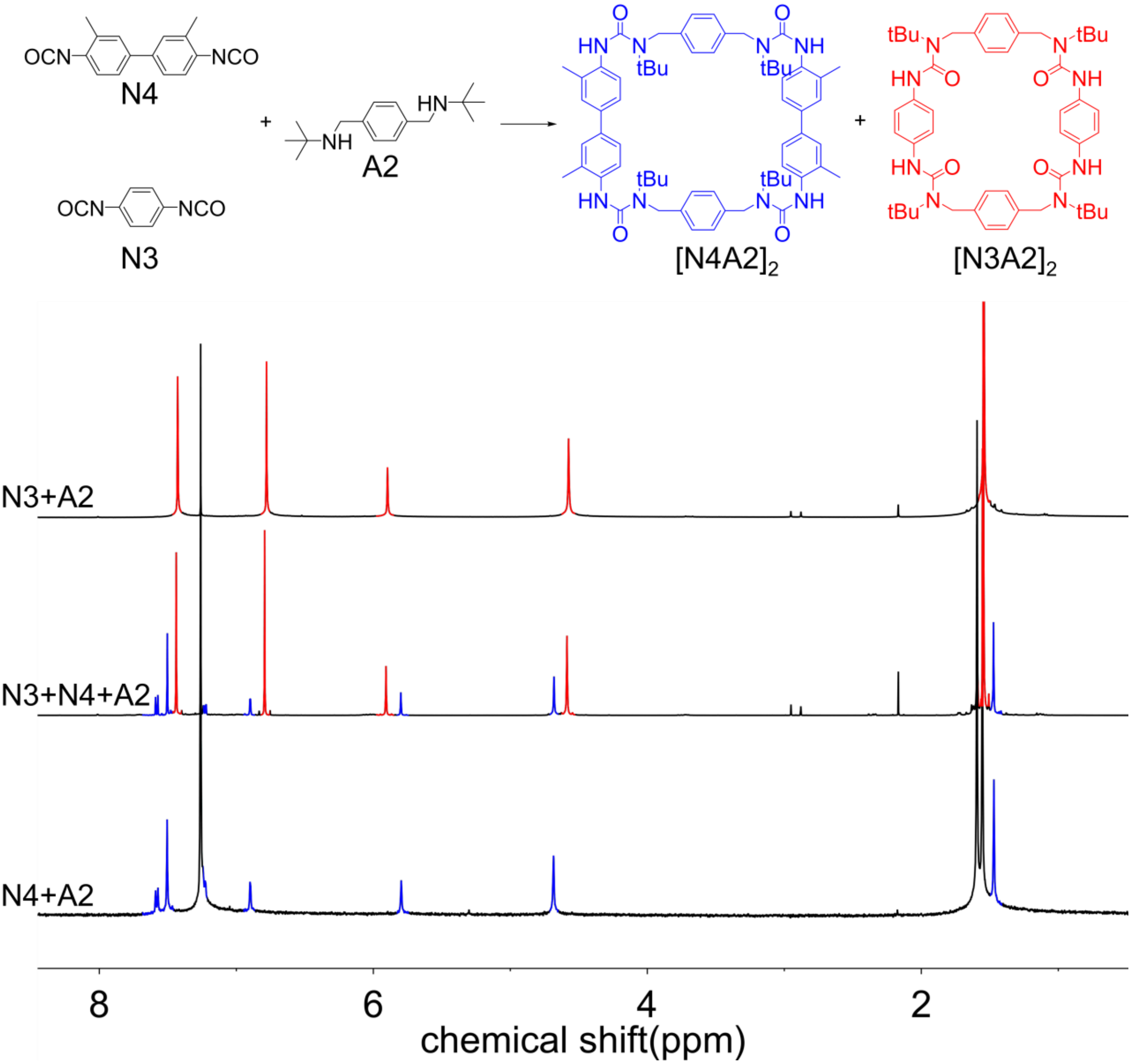

Supplementary Figure 28: ^{1}H NMR spectra showing the self-sorting behavior during HUM formation. When mixing two diisocyanate N3 and N4 with one diamine A2, $[N3A2]_2$ and $[N3A2]_2$ were formed in almost quantitative yields respectively with no [N3A2N4A2] or other hybrid species detected.

3.3. Validation for the role of the *tert*-butyl group

3.3.1. Synthesis of the model compounds

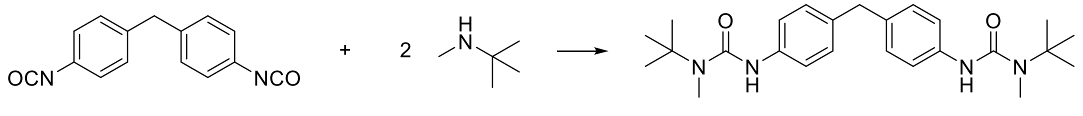

Supplementary Figure 29: Synthesis of **MC1**

Model compound 1 (MC1): Methylene diphenyl diisocyanate (125.0 mg, 0.5 mmol) and *N*-Methyl-*tert*-butylamine (87.2 mg, 1 mmol) were mixed in 10 mL DCM. The mixture was stirred at room temperature for 30 min and then solvent was removed. Compound 1 was obtained as white powder and used without purification. ^{1}H NMR (500 MHz, $CDCl_3$): δ 7.19 (d, J = 8.4 Hz, 4H), 7.05 (d, J = 8.5 Hz, 4H), 6.14 (s, 2H), 3.85 (s, 2H), 2.94 (s, 6H), 1.43 (s, 18H).

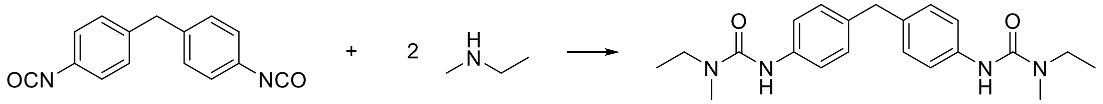

Supplementary Figure 30: Synthesis of **MC1’**

Model compound 1’ (MC1’): Methylene diphenyl diisocyanate (125.0 mg, 0.5 mmol) and *N*-Methylethylamine (59.1 mg, 1 mmol) were mixed in 10 mL DCM. The mixture was stirred at room temperature for 30 min and then solvent was removed. Compound 2 was obtained as white powder and used without purification. ^{1}H NMR (500 MHz, $CDCl_3$): δ 7.31 – 7.27 (m, 4H), 7.13 – 7.03 (m, 4H), 6.30 (s, 2H), 3.87 (s, 2H), 3.40 (q, J = 7.2 Hz, 4H), 2.99 (s, 6H), 1.19 (t, J = 7.2 Hz, 6H).

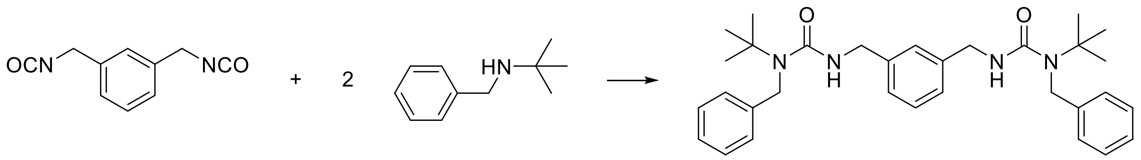

Supplementary Figure 31: Synthesis of **MC3**

Model compound 3 (MC3): m-Xylene diisocyanate (94.1 mg, 0.5 mmol) and *N*-(*Tert*-butyl)benzylamine (163.3 mg, 1 mmol) were mixed in 10 mL DCM. The mixture was stirred at room temperature for 30 min and then solvent was removed. MC3 was obtained as white powder and used without purification. ^{1}H NMR (500 MHz, $CDCl_3$): δ 7.33 (m, 6H), 7.25 – 7.20 (m, 4H), 7.12 (t, J = 7.6 Hz, 1H), 6.95 (dd, J = 7.7, 1.7 Hz, 2H), 6.85 (d, J = 1.8 Hz, 1H), 4.50 (s, 4H), 4.47 (d, J = 5.5 Hz, 2H), 4.25 (d, J = 5.5 Hz, 4H), 1.49 (s, 18H).

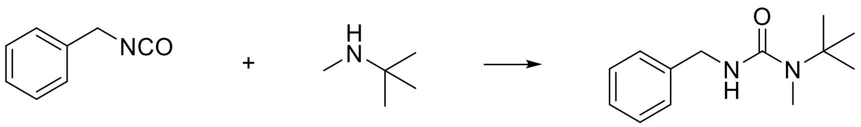

MC6

Supplementary Figure 32: Synthesis of **MC6**

MC6: Benzyl isocyanate (133.2 mg, 1 mmol) and *N*-Methyl-*tert*-butylamine (87.2 mg, 1 mmol) were mixed in 10 mL DCM. The mixture was stirred at room temperature for 30 min and then solvent was removed. Compound 3 was obtained as white powder and used without purification. ^{1}H NMR (500 MHz, $CDCl_3$): δ 7.32 (m, 5H), 4.39 (d, J = 5.4 Hz, 2H), 2.84 (s, 3H), 1.42 (s, 9H).

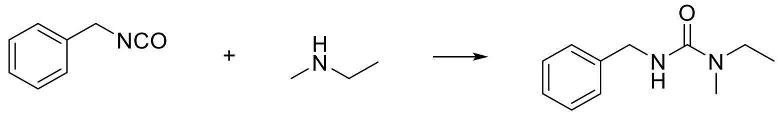

MC6'

Supplementary Figure 33: Synthesis of **MC6'**

MC6': Benzyl isocyanate (133.2 mg, 1 mmol) and *N*-Methylethylamine (59.1 mg, 1 mmol) were mixed in 10 mL DCM. The mixture was stirred at room temperature for 30 min and then solvent was removed. Compound 4 was obtained as white powder and used without purification. ^{1}H NMR (500 MHz, $CDCl_3$): δ 7.37 – 7.29 (m, 5H), 4.61 (s, 1H), 4.44 (d, J = 5.5 Hz, 2H), 3.33 (q, J = 7.1 Hz, 2H), 2.88 (d, J = 0.7 Hz, 3H), 1.12 (td, J = 7.1, 0.7 Hz, 3H).

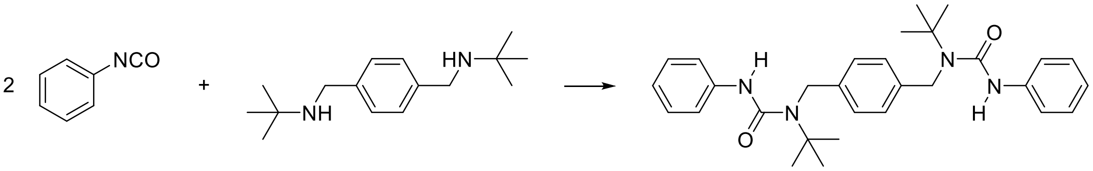

MC8

Supplementary Figure 34: Synthesis of **MC8**

Model compound 8 (MC8): Phenyl isocyanate (119.1 mg, 1 mmol) and the diamine A2 (124.2 mg, 0.5 mmol) were mixed in 10 mL DCM. The mixture was stirred at room temperature for 30 min and then solvent was removed. MC2 was obtained as white powder and used without purification. ^{1}H NMR (500 MHz, $CDCl_3$): δ 7.45 (s, 4H), 7.24 – 7.14 (m, 4H), 7.15 – 7.05 (m, 4H), 6.96 (t, J = 7.3 Hz, 2H), 6.17 (s, 2H), 4.65 (s, 4H), 1.54 (s, 18H).

3.3.2. NOE of the model compounds

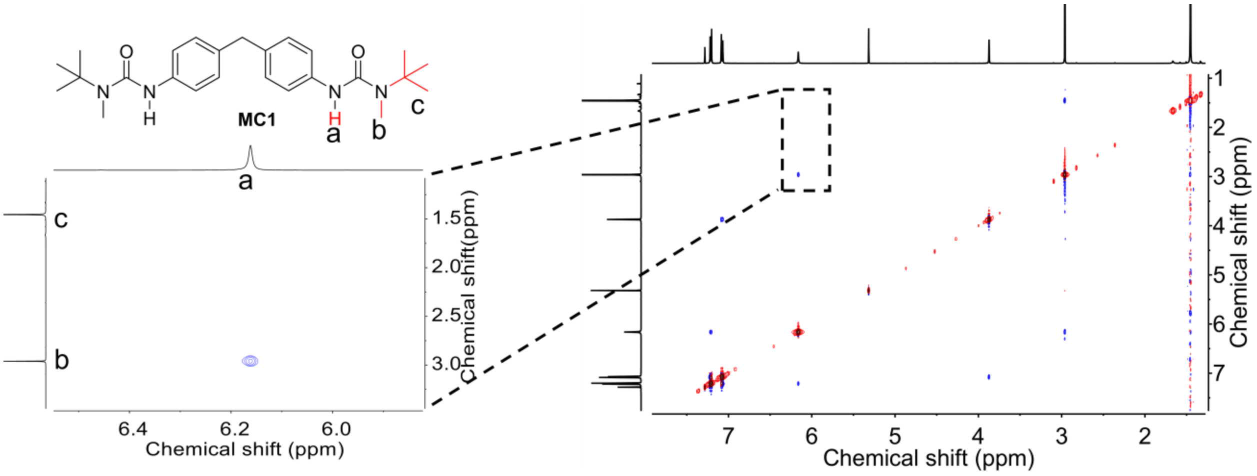

Supplementary Figure 35: NOE spectra of MC1. NH proton a is only in close proximity to the methyl group b but not to the t-Bu group c.

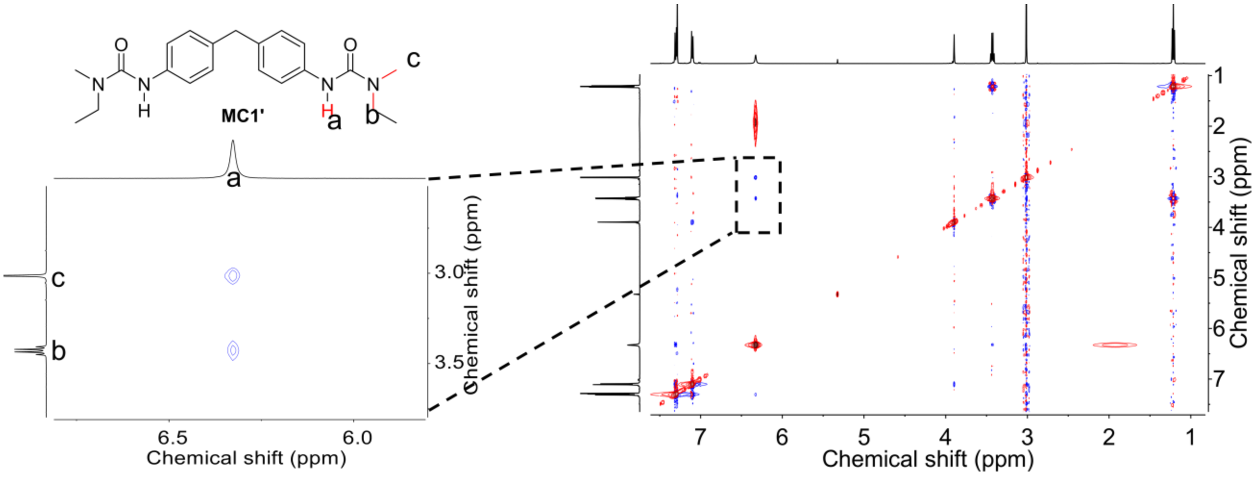

Supplementary Figure 36: NOE spectra of MC1'. NH proton a is in close proximity to both the ethyl group b and methyl group c.

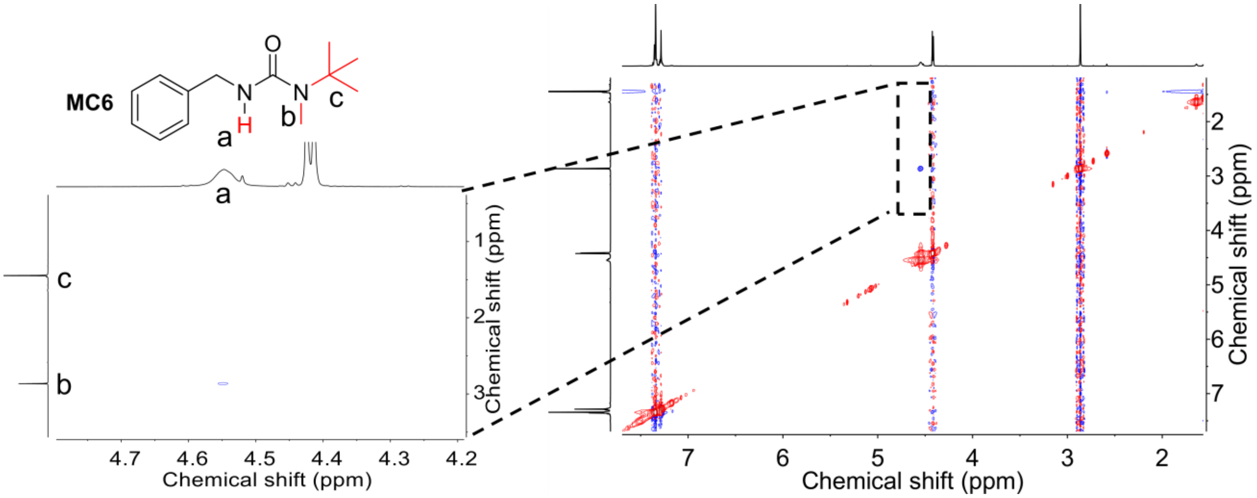

Supplementary Figure 37: NOE spectra of MC6. NH proton a is only in close proximity to the methyl group b but not to the t-Bu group c.

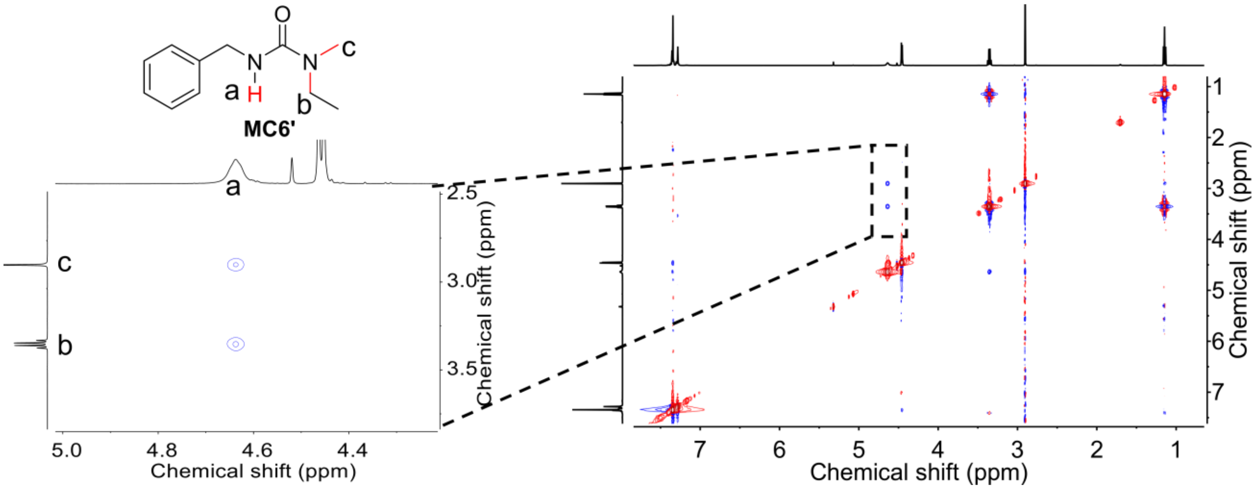

Supplementary Figure 38: NOE spectra of MC6'. NH proton a is in close proximity to both the ethyl group b and methyl group c.

3.3.3. DFT calculations

We performed quantum chemistry calculations using the Gaussian09 package to determine the relative structural interaction energies between cis and trans. To achieve high accuracy, calculations were performed with Density Functional Theory (DFT) and Møller-Plesset second order perturbation theory (MP2. For the DFT calculations, PBE functional at generalized gradient approximations (GGAs) level and Becke's three parameter hybrid exchange functional and Lee-Yang-Parr correlation functional (B3LYP) at hybrid level were selected. 6-31/G(d,p) basis set was used for both DFT functionals and aug-cc-pVDZ basis set was used for MP2. All calculations were performed at gas phase. Energies of two set of model compounds which vary only in one substituent and represent aliphatic HUBs (MC2 and MC2') and aromatic HUBs (MC7 and MC7') were calculated. The energy differences between *cis* and *trans* conformations (□$G_{trans\text{-}cis}$) were listed below.

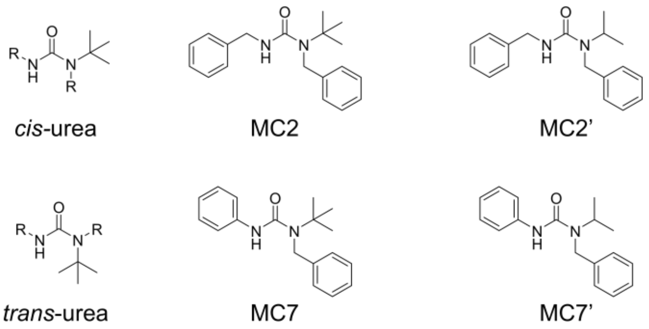

Compounds	$\Delta G_{trans\text{-}cis}$ (kcal/mol)		
	PBE(6-31G(d))	B3LYP(6-31G(d))	MP2
MC2	4.243090356	4.620093998	5.106867162
MC2'	1.264682266	1.354848093	1.901947776
MC7	4.877888501	5.141235203	5.600386676
MC7'	2.248301996	2.282626738	2.726368473

Supplementary Table 1: The energy differences between *cis* and *trans* conformations ($\Delta G_{trans\text{-}cis}$) of different model compounds.

The different methods all gave consistent results with the energies of the *cis* conformation being lower than the *trans* for both compounds. What's more, the energy differences between *cis/trans* conformations were much higher in MC2/7 than in MC2'/7', implying a much higher abundance of the '*cis*-urea' conformations and higher rotational barrier with the *t*-Bu substituent. Under the experimental condition, RT=0.663 kcal/mol. With tBu substituent, the energy difference between *cis* and *trans* were about 7 kT, meaning almost exclusive presence of the *cis*-urea conformation.

DFT calculations on different conformers of MC4

DFT calculations of the linear analogue MC4 (the [1:1] adduct of N2 and A2 with reactive chain ends) were performed at the PBE/6-31G(d) and B3LYP/6-31G(d) levels of theory. 12 different conformations were generated by rotating around the C(O)-N(*t*Bu) bond of the adduct, which converged into 5 different metastable conformations after local energy minimization. Relative free energy was defined as the energy difference with respect to most stable one. d was defined as the distance between the N of the free amine and C of the free isocyanate between which the reaction happens. θ1 was defined as the angle between vectors v1 and v2, which characterized the degree of folding of the urea chain. θ2 was defined as the angle between vectors v3 and v4, which characterized the position of the bulky *t*-Bu group with respect to the carbonyl group. Cis-conformation is defined as θ2 < 90°, while trans-conformation is defined as θ2 > 90°

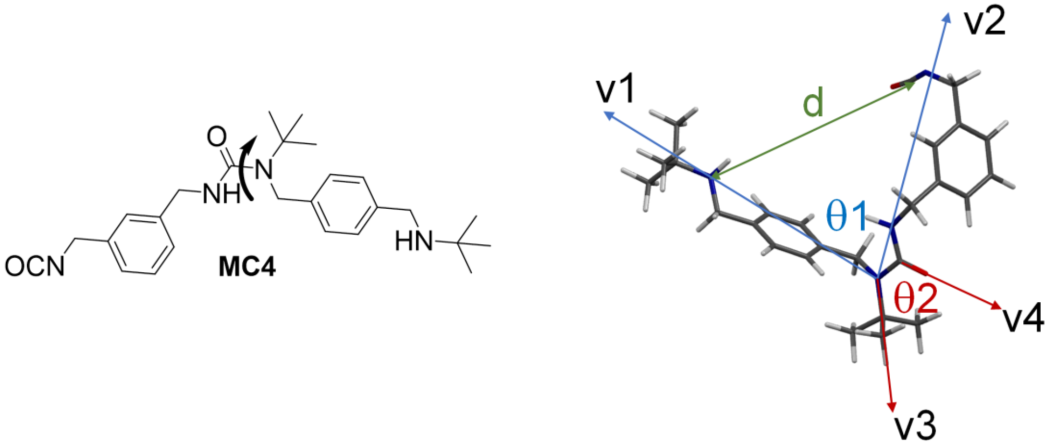

Supplementary Figure 39: structure of MC4 and illustration for the definition of different parameters.

Conformation	Relative energy (kcal/mol)	d (Å)	θ1	θ2
C1	0	4.460633	33.8	63.38
C2	2.1750	8.616266	68.6	63.21
C3	5.0157	10.422637	95.6	172.23
C4	6.3260	15.541224	154.2	173.12
C5	5.9279	15.049579	143.39	175.3

Supplementary Table 2: the calculated results of the relative energies, d, θ1 and θ2 for the five metastable conformations based on the B3LYP/6-31G(d) levels of theory.

Conformation	Relative energy (kcal/mol)	d (Å)	q1	q2
C1	0	4.433254	33.43	63.52
C2	2.627646	7.926678	61.41	63.27
C3	4.489096	10.247850	94.41	174.90
C4	5.781314	13.747061	118.60	175.15
C5	5.93602	14.962656	139.61	171.84

Supplementary Table 3: the calculated results of the relative energies, d, θ1 and θ2 for the five metastable conformations based on the PBE/6-31G(d) levels of theory.

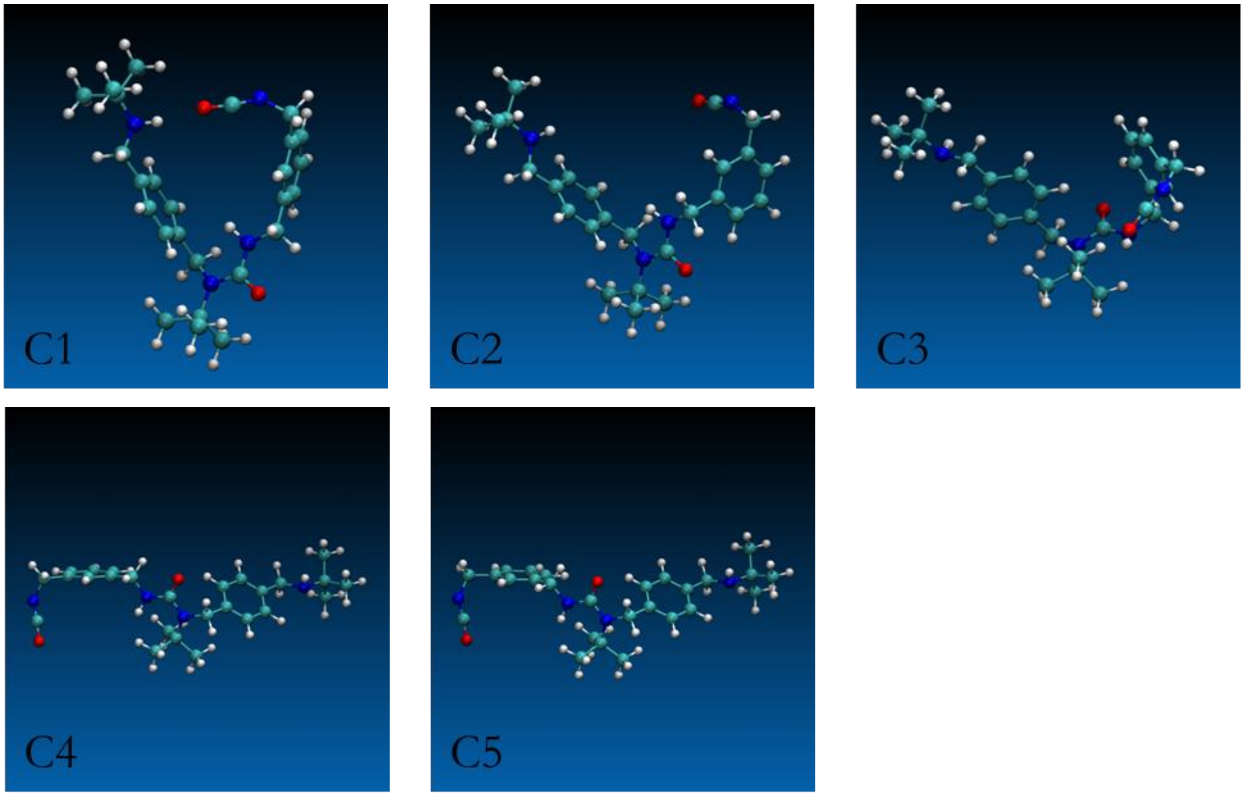

Supplementary Figure 40: structures of the five optimized conformations. Color code: C, green; H, white; O, red; N, blue.

3.3.4. Exchange reaction of two macrocycles and their model compounds

For the exchange reaction, 5 mg of each compound was dissolved in 600 □L $CDCl_3$ in a sealed NMR tube. The NMR instrument was set at 55 ºC and the sample was allowed to equilibrate in the instrument at 55 ºC for 5 min. The sample was then ejected and 20 □L butylisocyanate was quickly added to the tube and the sample was subjected to the NMR instrument immediately. Spectra were taken at various intervals. Remaining ratios were determined by the integration of the original peaks and new peaks. Since butylisocyanate was in large excess and its concentration can be regarded constant, the exchange reactions can be considered pseudo-first order.

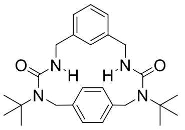

HUM2

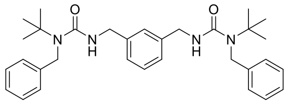

MC3

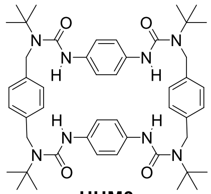

HUM3

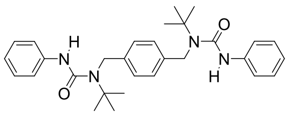

MC8

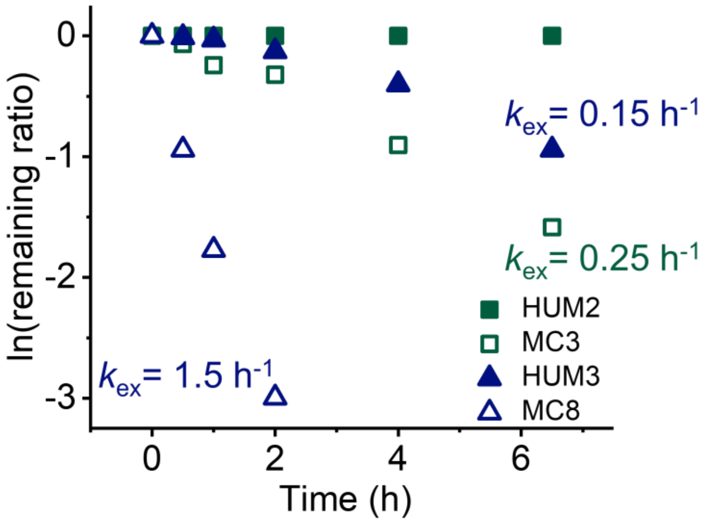

Supplementary Figure 41: Exchange kinetics of two macrocycles (HUM2 and HUM3) and their model compounds (MC3 and MC8) with butyl isocyanate in $CDCl_3$ at 55 ºC

Both macrocycles showed much slower exchange kinetics compared to their linear model compounds. What's more interesting, HUM2 seemed to be 'kinetically trapped'. It showed no exchange under the experimental condition and even after prolonged incubation of 48 h.

3.4. Role of t-Bu group in thermodynamic stabilization

3.4.1. Control experiment

We performed a similar reaction with only the t-Bu group in A1 changed to i-Pr (A1'). In this case, only a mixture of oligomeric molecules was obtained even with prolonged incubation time. Although it is known that A1' based hindered ureas are less dynamic than A1 based ones, our previous work has shown that the k_{-1} of the corresponding urea structure is still decent for the mixture to reach its thermodynamically stable state under mild conditions. The mixture was proved to reach chemical equilibrium after 15 days without production of exclusive macrocyclic products.

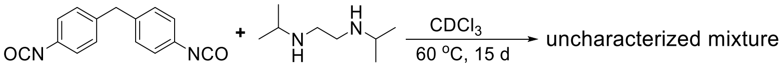

N1 **A1'**

Control reaction: Methylene diphenyl diisocyanate (N1, 25 mg, 0.1 mmol) was dispersed in $CDCl_3$ (1 mL) and sonicated. *N,N'*-Di-isopropylethylenediamine (A1', 14.4 mg, 0.1 mmol) in 1 mL $CDCl_3$ was added to the solution of N1. The mixture was capped and incubated at 60 °C for 15 days. Only an uncharacterized mixture was observed throughout the process. To exclude the possibility of hydrolysis, the reaction was also repeated in glovebox with 4 Å MS added and $CDCl_3$ pre-dried. No change of result was observed.

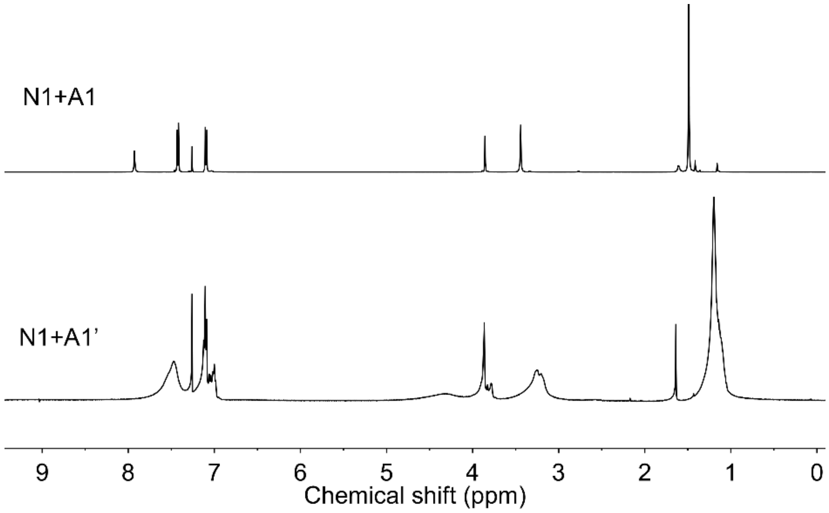

Supplementary Figure 42: Comparison of the 1H NMR spectra (500 MHz) of N1+A1 and N1+A1' at equilibration states (60 °C, 50 mM in $CDCl_3$).

3.4.2. Concentration dependent NMR and NOE studies

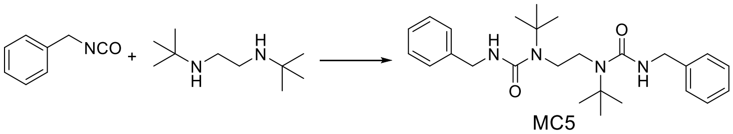

Supplementary Figure 43: Synthesis of **MC5**

Model compound 5 (MC5): Benzyl isocyanate (133.1 mg, 1 mmol) and *N,N′*-Di-*tert*-butylethylenediamine (86.2 mg, 0.5 mmol) were mixed in 10 mL DCM. The mixture was stirred at room temperature for 30 min and then solvent was removed. MC2 was obtained as white powder and used without purification. ^{1}H NMR (500 MHz, $CDCl_3$): δ 7.29 (m, 8H), 7.23 (m, 2H), 6.32 (t, J = 5.6 Hz, 2H), 4.35 (d, J = 5.5 Hz, 4H), 3.32 (s, 4H), 1.34 (s, 18H).

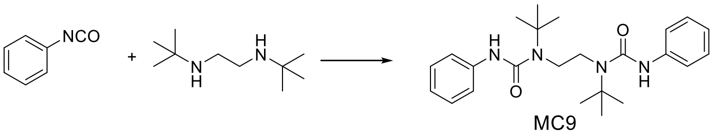

Supplementary Figure 44: Synthesis of **MC9**

Model compound 9 (MC9): Phenyl isocyanate (119.1 mg, 1 mmol) and *N,N′*-Di-*tert*-butylethylenediamine (86.2 mg, 0.5 mmol) were mixed in 10 mL DCM. The mixture was stirred at room temperature for 30 min and then solvent was removed. MC1 was obtained as white powder and used without purification. ^{1}H NMR (500 MHz, $CDCl_3$): δ 7.97 (s, 2H), 7.54 (m, 4H), 7.28 (m, 4H), 7.02 (tt, J = 7.3, 1.1 Hz, 2H), 3.50 (s, 4H), 1.53 (s, 18H).

Hindered urea macrocycle 1 (HUM1): Methylene diphenyl diisocyanate (N1, 250 mg, 1 mmol) was dispersed in THF (5 mL) and sonicated. *N,N′*-Di-*tert*-butylethylenediamine (A1, 172 mg, 1 mmol) in 5 mL THF was added to the solution of N1. White needle-like crystals were quickly formed. The mixture was capped and incubated at 60 ^oC overnight. The mixture was filtered and the white precipitates (HUM1) were collected and used for further studies.

Concentration dependent NMR: HUM1 and MC9 were dissolved in $CDCl_3$ and diluted to make the final concentrations 1mM, 5 mM, 20 mM and 50 mM respectively. ^{1}H NMR were taken and the peak shifts were monitored. For the macrocycle HUM1, when the concentration goes up, the *tert*-butyl peak shifted upfield while other peaks showed negligible changes, including the NH proton. For its linear model compound, all peaks showed negligible changes.

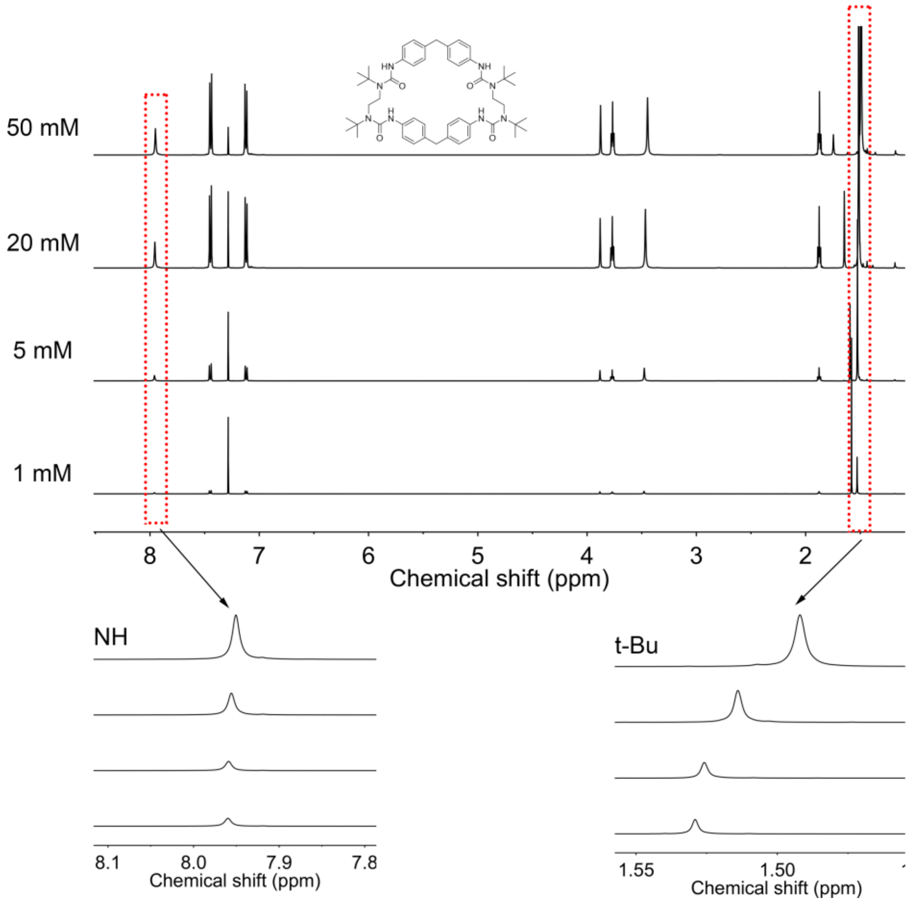

Supplementary Figure 45: Concentration dependent NMR of HUM1 (500 MHz, $CDCl_3$). The NH peak and *t*-Bu peak were enlarged for clarity.

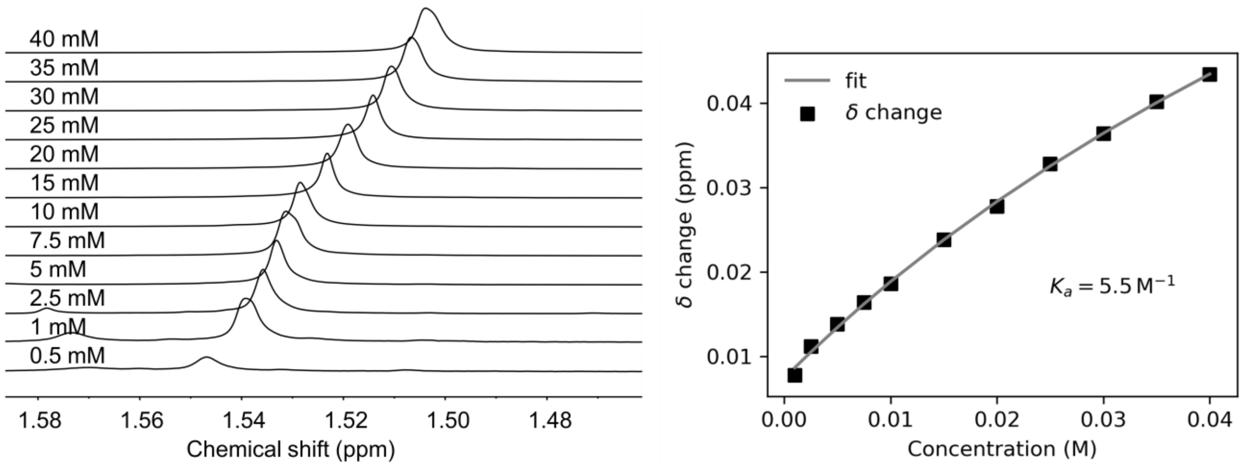

Supplementary Figure 46: Determination of dimeric association constant of HUM1 in CDCl3. (500 MHz, 298 K).

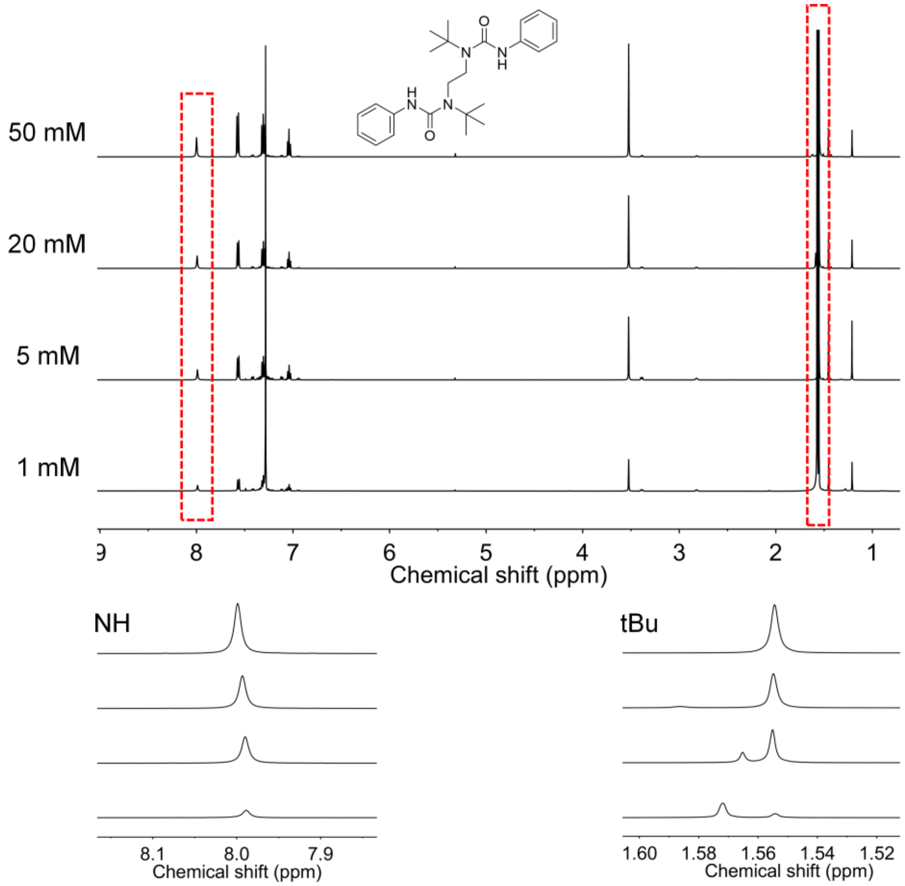

Supplementary Figure 47: Concentration dependent NMR of MC9 (500 MHz, $CDCl_3$). The NH peak and *t*-Bu peak were enlarged for clarity.

NOE study: Firstly the ^{1}H NMR of a mixture of HUM1 (25 mM) and MC5 (50 mM) in CDCl3 was taken on an Agilent VNS 750NB spectrometer. The peaks of them were resolved and then the *t*-Bu peak of HUM1 was irradiated at 1.47 ppm with a selective band width of 13.2 Hz. The NOE peaks were recorded.

3.4.3. Simulation results

To further prove our hypothesis that t-Bu···macrocycle interactions contribute to the high yield, atomistic level molecular simulation was performed to calculate the average monomeric non-bonding energy in macrocycles with various sizes n (denoted as n-mer), either in single macrocycles states (denoted as nmer-s) or in multiple macrocycles clusters (denoted as nmer-m). A thermodynamic model which utilizes the computed monomeric energies was proposed to compute the stable ring size distribution which minimizes the system free energy (see part 2). For N1+A1 reaction, the average monomeric energy computed in 1mer, 2mer and 3mer are shown in (**Supplementary Figure 48a**), these energies are then plugged into the thermodynamics model to predict the stable distribution. When inter-macrocycle interactions were omitted (nmer-s system), there is coexistence of 2mer, 3mer and 4mer, with 3mer being the major species (**Supplementary Figure 48b**). However, when the interactions between macrocycles were considered (nmer-m system), the corresponding size distribution showed the predominance of 2mer alone, which was consistent with the experimental results. This implies the interactions between macrocycles stabilize the 2mers and drive it to be the much more favored species. No such effect was observed in the control N1+A1' system.

To further investigate the mode of interactions between the macrocycles, dimerization potential of mean force (PMF) was computed between two 2mer and 3mer macrocycles (**Supplementary Figure 48c**) using umbrella sampling technique; 1mer system was not included since it has extremely high ring strain which overwhelms the intermolecular interactions. The normalized stabilization energy is defined as (*PMF well depth*)/n. 2mer showed a higher stabilization free energy (4.0 kT) than 3mers (2.5 kT) (**Supplementary Figure 48c**, inset). To see how the macrocycles interact with each other, the motions of the macrocycles were tracked around the well region where the center of mass distance of two rings is about 0.8 nm. For the 2mer system, a "pocket effect" was clearly observed, with *t*-Bu group from one macrocycle sitting in the cavity of another one, which lower the stabilization energy and was in consistence with the structure in the solid state (**Supplementary Figure 48e**). In contrast, for the 3mer system, no clear 'pocket effect' was observed. This was further supported by the probability density distribution of the distance between *t*-Bu groups and the 'pocked center' of the other macrocycle (**Supplementary Figure 48d**). In the 2mer system, the t-Bu group has a much higher probability to appear very close to the pocket center of the other macrocycle, signaling the existence of the extra interaction between t-Bu --- macrocycle. In the 3mer system, the distribution is more scattered and does not show an obvious preference.

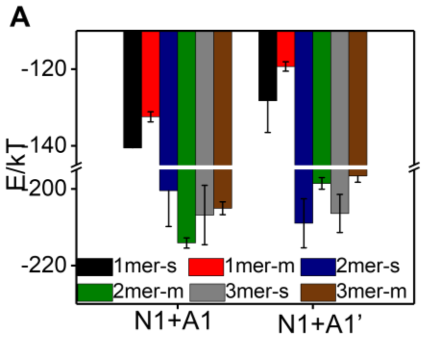

B

Mass fraction						
Ring size	1	2	3	4	5	6
E_s	<E-5	0.031	0.968	0.001	<E-5	<E-5
E_m	<E-5	1	<E-5	<E-5	<E-5	<E-5

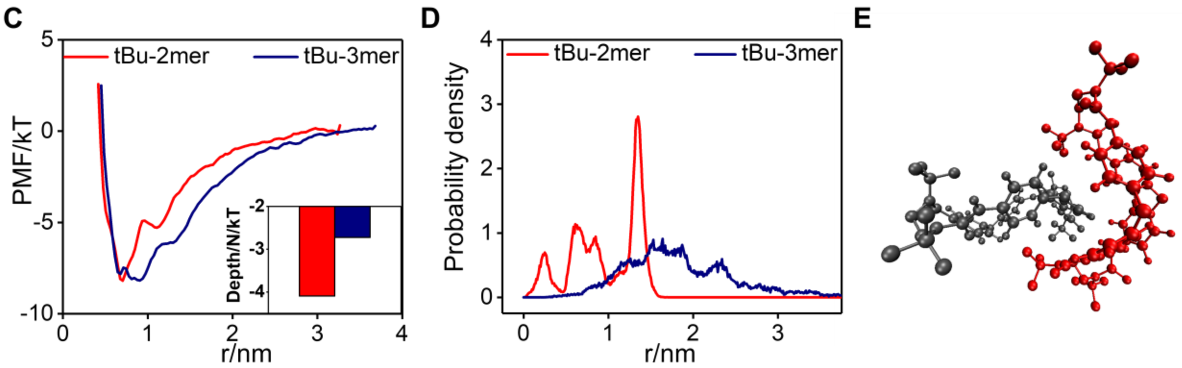

Supplementary Figure 48: a Average monomeric energy of N1+A1 and the control N1+A1’ system; number means size of the ring, ‘s’ only considers the energy of a single ring, ‘m’ includes interactions between rings. For N1+A1, The energy of 2mer-m system is lower because of ring-ring interaction. While for N1+A1’ system, ring-ring interactions did not result in energy drop. **b** Calculated size distribution of the tBu system based on E_s and E_m. **c** Dimerization potential of mean force (PMF) between two 2mer and 3mer macrocycles. Inset: normalized well depth **d** Probability density distribution of the distance between *tert*-butyl group and the pocket center of the other ring around the well of the PMF curve for for different sized rings in both t-Bu and i-Pr systems. **e** Simulated mode of interactions of two HUM1 in the PMF well region.

3.5 De-*tert*-butylation

3.5.1. Self-assembled fiber of UM1

General procedure for de-*tert*-butylation: The HUMs were treated with TFA for 5 min at room temperature. Then the TFA was removed under vacuo. The solid was washed with diluted $NaHCO_3$ solution, water, Acetone and then dried under vacuo.

Self-assembled structure of UM1:

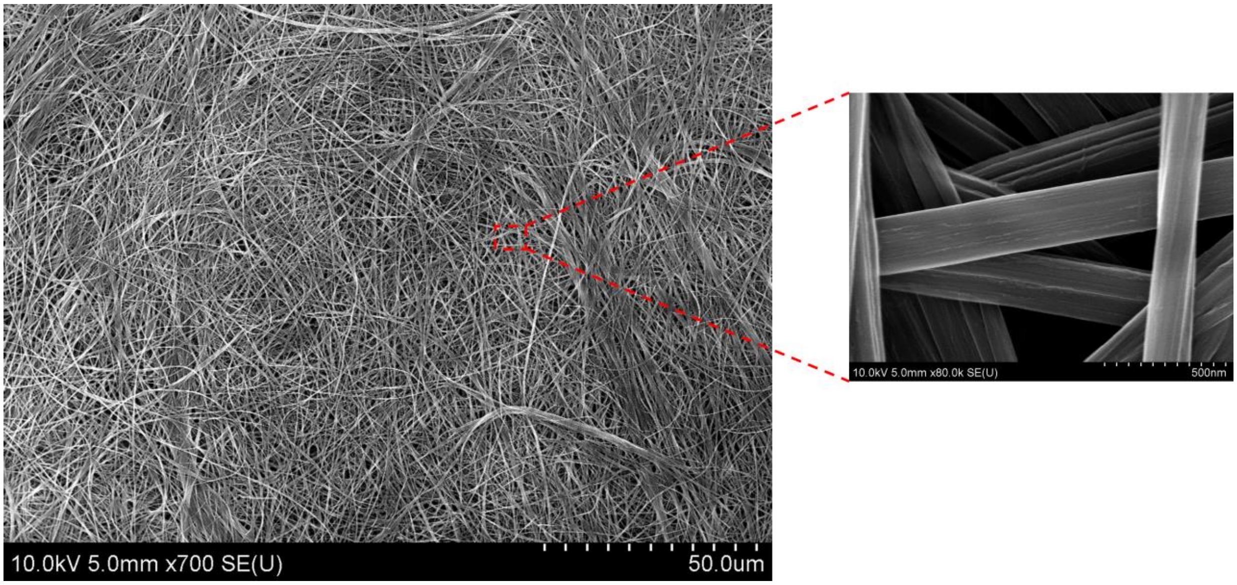

Supplementary Figure 49: SEM image of the self-assembled structure of UM1.

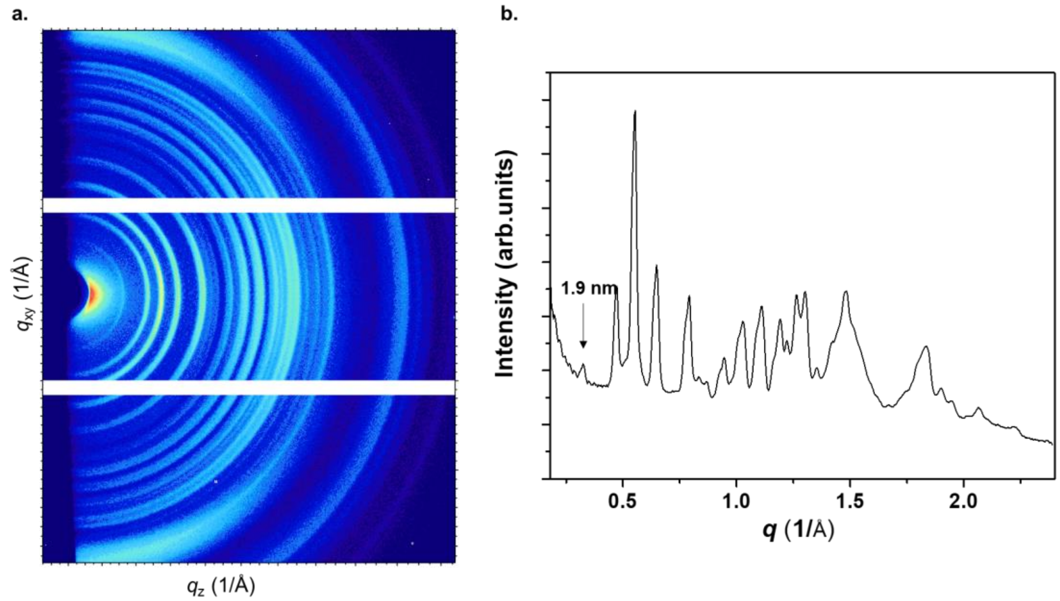

c.

q (1/Å)	d (Å)
0.3237	19.41
0.4733	13.28
0.5542	11.34
0.6472	9.71
0.7928	7.93
0.8332	7.54
0.8696	7.23
0.9464	6.64
1.0273	6.12
1.1122	5.65
1.1931	5.27
1.2214	5.14
1.2619	4.98
1.3023	4.82
1.3508	4.65
1.4843	4.23
1.6703	3.76
1.8321	3.43
1.9	3.31
1.9453	3.23
2.0626	3.05
2.2203	2.83

Supplementary Figure 50: Grazing-Incidences Wide-Angle X-ray Scattering (GIWAXS) of UM1. (a) 2D X-ray scattering GIWAXS image. (b) 1D integrated intensity versus q. (c) the size scale of the real space ordering corresponding to the peaks in b. 1.9 nm is possibly the pacing distance between fibrils.

3.5.2. Binding of UM1 with anions

Urea macrocycles have been shown to be potent anion binders. As a proof of concept, two urea macrocycles obtained by the coupling of HUB chemistry and acid-assisted de-*tert*-butylation, UM1 and UM2 were tested for their anion binding ability. UM1 showed selective binding to the organic salts such as phosphates and acetates and no interaction to halides while UM2 showed size-selective binding to different halides.

To determine the binding constant, UM1 and tetrabutylammonium phosphate monobasic were chosen as the model system. The concentrations of the host molecule UM1 were kept constant and different equivalents of guest tetrabutylammonium phosphates were added. NMR were taken to monitor the chemical shift changes.

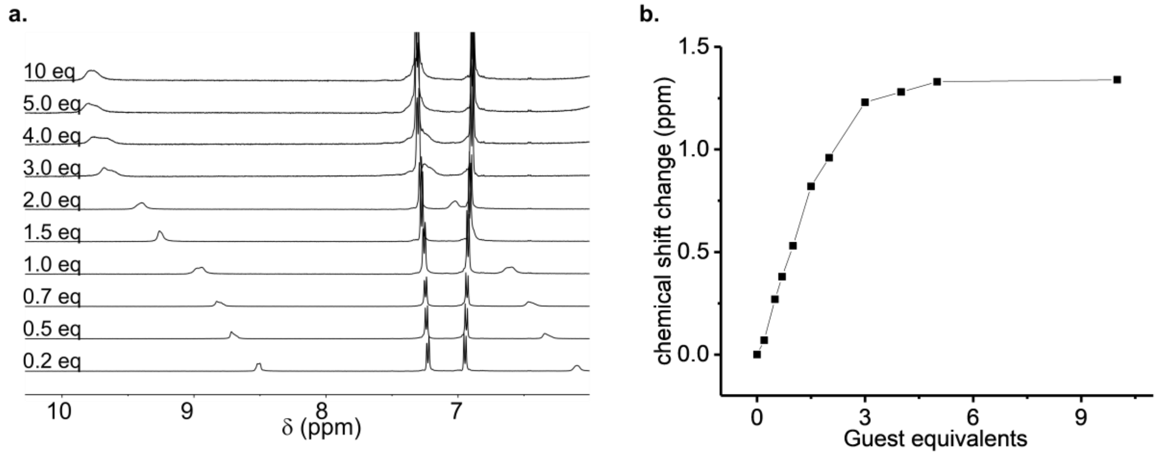

Supplementary Figure 51: Binding test between UM1 and tetrabutylammonium phosphate monobasic (TBAP). (a) NMR spectra showing the chemical shift changes of UM1 with the addition of different equivalents of TBAP in DMSO-d6. Concentration of UM1 is 10 mM. (b) Chemical shift changes of UM1 with the addition of different equivalents of TBAP. Binding constant (Ka ~ 53000 M^{-1}) was obtained by non-linear fit.

3.5.3. Synthesis of the De-[N2A4]-C12 and its antimicrobial activity test

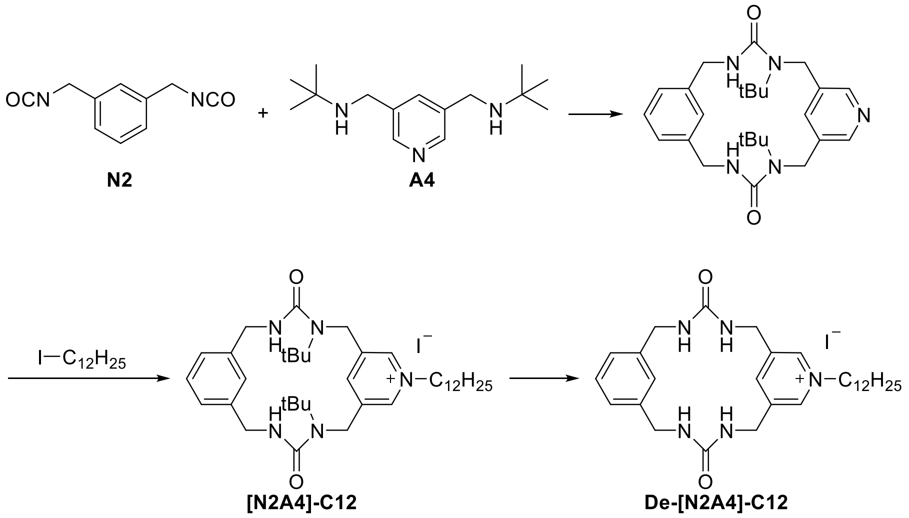

Supplementary Figure 52: Synthesis of **De-[N2A4]-C12**

De-[N2A4]-C12: N2 (94.1 mg, 0.5 mmol) and A4 (124.7 mg, 05 mmol) were mixed in 10 mL ethyl acetate at stirred at 75 °C overnight. After confirmation of complete transformation to the macrocycle species $[N2A4]_1$ by TLC (only one spot on the TLC plate), 1-Iodododecane (592.5 mg, 2 mmol) was added. The mixture was stirred at 75 °C overnight and the alkylated macrocycles gradually precipitated out. After confirmation of no free macrocycle remaining by TLC, the mixture was concentrated and the precipitates were collected by filtration and then washed by Hexane. The solid was then treated with standard de-*tert*-butylation procedures. Final products were obtained as yellow solid. ^{1}H NMR (500 MHz, DMSO-d6) δ 8.92 (s, 2H), 8.27 (s, 1H), 7.18 (td, J = 7.5, 1.7 Hz, 1H), 7.01 (d, J = 7.6 Hz, 2H), 6.96 (s, 1H), 6.80 (t, J = 5.9 Hz, 2H), 4.79 (s, 4H), 4.57 (t, J = 7.5 Hz, 2H), 4.29 (d, J = 5.6 Hz, 4H), 1.92 (s, 2H), 1.35 (d, J = 1.7 Hz, 18H), 1.25 (d, J = 19.6 Hz, 18H), 0.91 – 0.77 (m, 3H).

Antimicrobial test: three different strains of Methicillin-resistant Staphylococcus aureus (MRSA) USA100, USA200 and USA300 were grown in LB medium at 37 °C. For determination of the MIC, the sample De-[N2A4]-C12 was dissolved in media using serial dilutions from a stock solution. Vancomycin was used as positive control. Into each well of a 96-well plate was added 200 µL of each concentration and 2 µL of bacteria (1 × 10^8 CFU (colony forming units)) in medium. The plate was incubated at 37 °C. The optical density readings of microorganism solutions at 600 nm were measured after 24 h incubation. The MIC was considered as the lowest concentration of peptide where no visual growth of bacteria was detected.

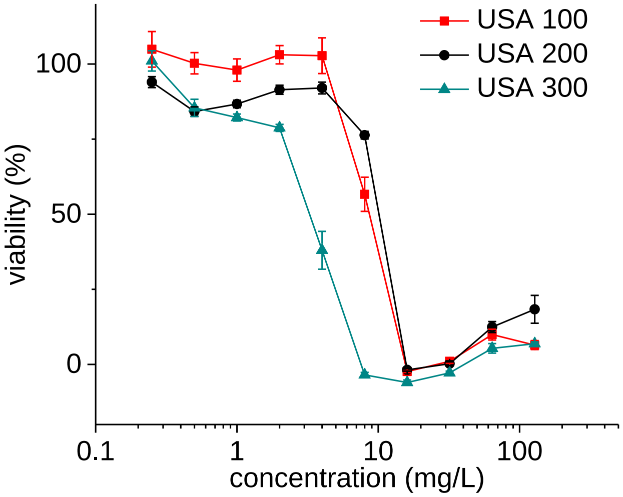

Supplementary Figure 53: Antimicrobial activity of De-[N2A4]-C12 against three different strains of MRSA.

4. Crystallographic Data

Singles crystals of HUM1 were grown by slow evaporation of a solution of HUM1 in a mixture of THF and Acetonitrile. The crystals diffracted very weakly due to the lack of heavy atoms and disordered solvents within the cavity. Intensity data were collected on a Bruker D8 Venture kappa diffractometer equipped with a Photon 100 CMOS detector. An Iμs microfocus source provided the Cu Kα radiation (λ = 1.54178 Å) that was monochromated with multilayer mirrors. The collection, cell refinement and integration of intensity data was carried out with the APEX2 software (15). Face-indexed absorption corrections were performed numerically with SADABS (16). The structures were solved with the intrinsic phasing methods SHELXT (17). All structure were refined with the full-matrix least-squares SHELXL program. Analysis of the available data results in a chemically reasonable structure model that confirms the target molecule was synthesized.

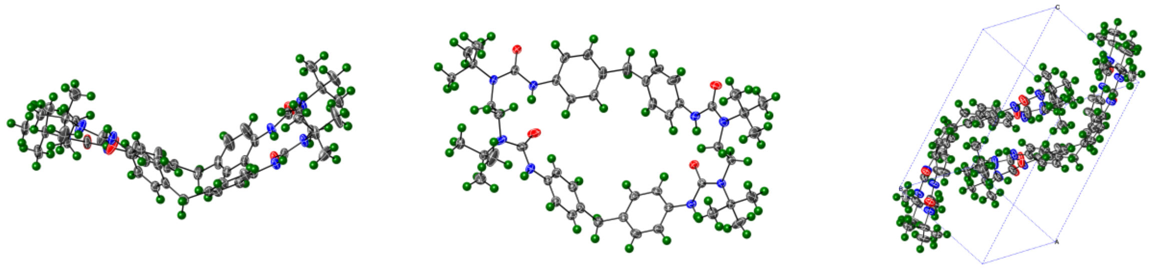

Supplementary Figure 54: Single crystal structure of HUM1. Left: side view; middle: top view; right: packing. Color code: C, grey; N, blue; O, red; H, green.

Supplementary Table 4. Crystal data and structure refinement for dd12is.

Identification code	dd12is	
Empirical formula	C56.68 H80.70 N8.66 O5.34	
Formula weight	968.83	
Temperature	100(2) K	
Wavelength	1.54178 Å	
Crystal system	Triclinic	
Space group	P-1	
Unit cell dimensions	a = 11.4754(3) Å	α= 87.3318(9)°.
	b = 15.0276(4) Å	β= 89.0386(10)°.
	c = 16.2586(4) Å	γ = 70.5058(9)°.
Volume	2640.17(12) $Å^3$	
Z	2	
Density (calculated)	1.219 Mg/m^3	
Absorption coefficient	0.626 mm^{-1}	
F(000)	1048	
Crystal size	0.308 x 0.219 x 0.156 mm^3	
Theta range for data collection	2.721 to 68.380°.	
Index ranges	-13<=h<=13, -17<=k<=18, -19<=l<=18	
Reflections collected	26888	
Independent reflections	9527 [R(int) = 0.0438]	
Completeness to theta = 67.679°	98.6 %	
Absorption correction	Semi-empirical from equivalents	
Max. and min. transmission	0.7531 and 0.6735	
Refinement method	Full-matrix least-squares on F^2	
Data / restraints / parameters	9527 / 361 / 776	
Goodness-of-fit on F^2	1.054	
Final R indices [I>2sigma(I)]	R1 = 0.0772, wR2 = 0.1867	
R indices (all data)	R1 = 0.0907, wR2 = 0.1979	
Extinction coefficient	0.0032(3)	
Largest diff. peak and hole	0.915 and -0.779 e.$Å^{-3}$	

Singles crystals of HUM3 ([N3A2]$_2$) were grown by slow evaporation of a solution of HUM2 in a mixture of chloroform and hexane. The crystals diffracted very weakly due to the lack of heavy atoms and disordered solvents within the cavity. Intensity data were collected on a Bruker D8 Venture kappa diffractometer equipped with a Photon 100 CMOS detector. An Iμs microfocus source provided the Cu Kα radiation (λ = 1.54178 Å) that was monochromated with multilayer mirrors. The collection, cell refinement and integration of intensity data was carried out with the APEX2 software (15). Face-indexed absorption corrections were performed numerically with SADABS (16). The structures were solved with the intrinsic phasing methods SHELXT (17). All structure were refined with the full-matrix least-squares SHELXL program. Analysis of the available data results in a chemically reasonable structure model that confirms the target molecule was synthesized.

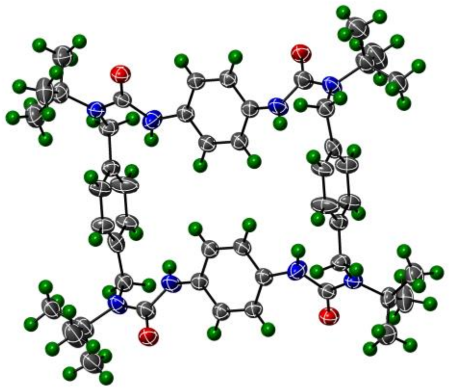

Supplementary Figure 55: Single crystal structure of HUM3. Color code: C, grey; N, blue; O, red; H, green.

Supplementary Table 5. Crystal data and structure refinement for dd95gs_sq.

Identification code	dd95gs_sq	
Empirical formula	C48 H64 N8 O4	
Formula weight	817.07	
Temperature	225(2) K	
Wavelength	1.54178 Å	
Crystal system	Triclinic	
Space group	P-1	
Unit cell dimensions	a = 19.2748(10) Å	α= 108.976(3)°.
	b = 20.1404(10) Å	β= 95.541(2)°.
	c = 20.5289(11) Å	γ = 112.994(2)°.
Volume	6704.8(6) $Å^3$	
Z	4	
Density (calculated)	0.809 Mg/m^3	
Absorption coefficient	0.415 mm^{-1}	
F(000)	1760	
Crystal size	0.495 x 0.180 x 0.138 mm^3	
Theta range for data collection	2.355 to 50.760°.	
Index ranges	-19<=h<=19, -20<=k<=20, -20<=l<=20	
Reflections collected	40825	
Independent reflections	14084 [R(int) = 0.0715]	
Completeness to theta = 50.760°	98.8 %	
Absorption correction	Semi-empirical from equivalents	
Max. and min. transmission	0.7500 and 0.3994	
Refinement method	Full-matrix least-squares on F^2	
Data / restraints / parameters	14084 / 1321 / 1043	
Goodness-of-fit on F^2	2.389	
Final R indices [I>2sigma(I)]	R1 = 0.2061, wR2 = 0.5339	
R indices (all data)	R1 = 0.2312, wR2 = 0.5635	
Extinction coefficient	n/a	
Largest diff. peak and hole	0.932 and -0.852 e.$Å^{-3}$	

Singles crystals of $[N4A2]_2$ were grown by slow evaporation of a solution of $[N4A2]_2$ in a mixture of chloroform and hexane. The crystals diffracted very weakly due to the lack of heavy atoms and disordered solvents within the cavity. Intensity data were collected on a Bruker D8 Venture kappa diffractometer equipped with a Photon 100 CMOS detector. An Iμs microfocus source provided the Cu Kα radiation (λ = 1.54178 Å) that was monochromated with multilayer mirrors. The collection, cell refinement and integration of intensity data was carried out with the APEX2 software (15). Face-indexed absorption corrections were performed numerically with SADABS (16). The structures were solved with the intrinsic phasing methods SHELXT (17). All structure were refined with the full-matrix least-squares SHELXL program. Analysis of the available data results in a chemically reasonable structure model that confirms the target molecule was synthesized.

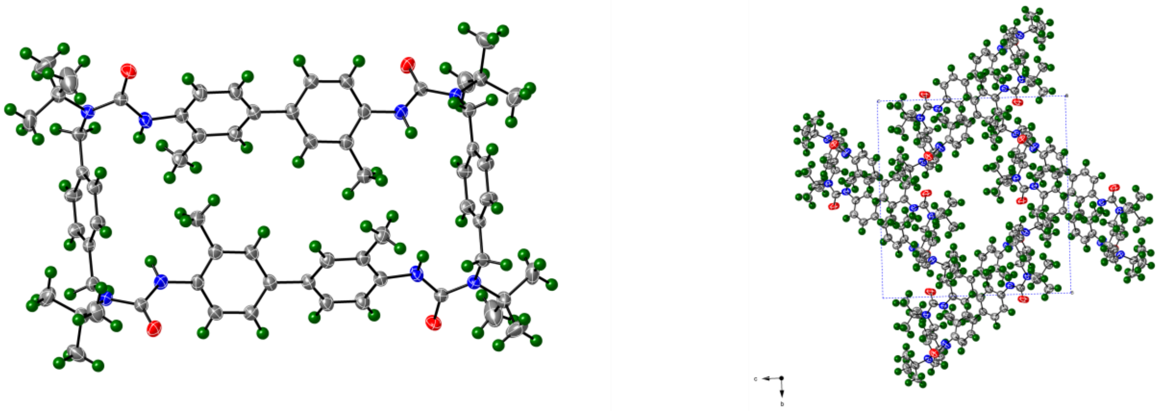

Supplementary Figure 56: Single crystal structure of $[N4A2]_2$. Left: top view; right: packing. Color code: C, grey; N, blue; O, red; H, green.

Supplementary Table 6. Crystal data and structure refinement for dd96gs_Sq.

Identification code	dd96gs_sq	
Empirical formula	C64 H80 N8 O4	
Formula weight	1025.36	
Temperature	225(2) K	
Wavelength	1.54178 Å	
Crystal system	Monoclinic	
Space group	$P2_1/c$	
Unit cell dimensions	a = 10.2515(4) Å	α= 90°.
	b = 19.4696(7) Å	β= 94.5975(13)°.
	c = 18.6527(7) Å	γ = 90°.
Volume	3711.0(2) $Å^3$	
Z	2	
Density (calculated)	0.918 Mg/m^3	
Absorption coefficient	0.453 mm^{-1}	
F(000)	1104	
Crystal size	0.667 x 0.125 x 0.068 mm^3	
Theta range for data collection	3.287 to 68.414°.	
Index ranges	-12<=h<=12, -23<=k<=23, -22<=l<=22	
Reflections collected	57967	
Independent reflections	6746 [R(int) = 0.0623]	
Completeness to theta = 67.679°	98.8 %	
Absorption correction	Semi-empirical from equivalents	
Max. and min. transmission	0.7531 and 0.4209	
Refinement method	Full-matrix least-squares on F^2	
Data / restraints / parameters	6746 / 0 / 362	
Goodness-of-fit on F^2	1.309	
Final R indices [I>2sigma(I)]	R1 = 0.0487, wR2 = 0.1623	
R indices (all data)	R1 = 0.0601, wR2 = 0.1719	
Extinction coefficient	n/a	
Largest diff. peak and hole	0.322 and -0.262 e.$Å^{-3}$	

Singles crystals of MC5 were grown by slow evaporation of a solution of MC5 in a mixture of chloroform and hexane. The crystals diffracted very weakly due to the lack of heavy atoms and disordered solvents within the cavity. Intensity data were collected on a Bruker D8 Venture kappa diffractometer equipped with a Photon 100 CMOS detector. An Iμs microfocus source provided the Cu Kα radiation (λ = 1.54178 Å) that was monochromated with multilayer mirrors. The collection, cell refinement and integration of intensity data was carried out with the APEX2 software (15). Face-indexed absorption corrections were performed numerically with SADABS (16). The structures were solved with the intrinsic phasing methods SHELXT (17). All structure were refined with the full-matrix least-squares SHELXL program. Analysis of the available data results in a chemically reasonable structure model that confirms the target molecule was synthesized.

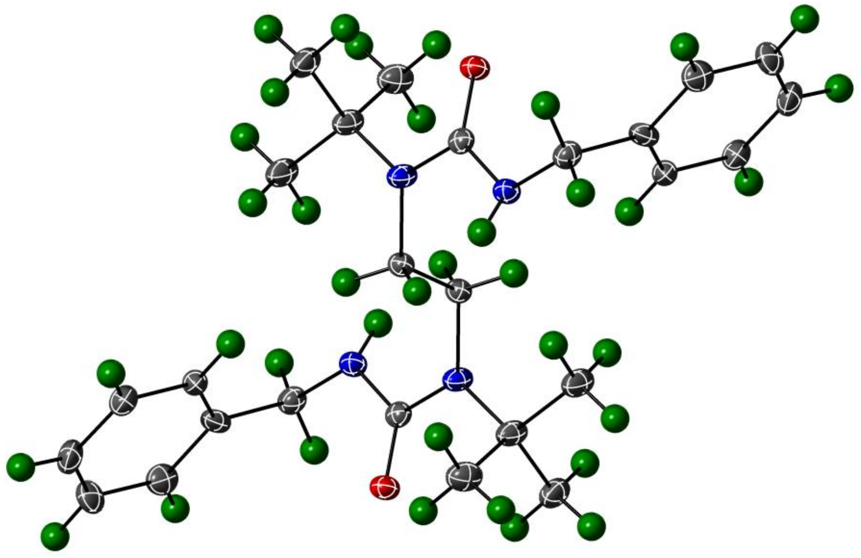

Supplementary Figure 57: Single crystal structure of MC5. Left: top view; right: packing. Color code: C, grey; N, blue; O, red; H, green.

Supplementary Table 7. Crystal data and structure refinement for dd53is.

Identification code	dd53is	
Empirical formula	C26 H38 N4 O2	
Formula weight	438.60	
Temperature	100(2) K	
Wavelength	0.71073 Å	
Crystal system	Monoclinic	
Space group	$P2_1/c$	
Unit cell dimensions	a = 11.4207(4) Å	α= 90°.
	b = 11.8485(4) Å	β= 96.2773(11)°.
	c = 9.4122(3) Å	γ = 90°.
Volume	1266.01(7) $Å^3$	
Z	2	
Density (calculated)	1.151 Mg/m^3	
Absorption coefficient	0.074 mm^{-1}	
F(000)	476	
Crystal size	0.233 x 0.179 x 0.152 mm^3	
Theta range for data collection	2.774 to 25.374°.	
Index ranges	-13<=h<=13, -14<=k<=14, -11<=l<=11	
Reflections collected	20118	
Independent reflections	2319 [R(int) = 0.0302]	
Completeness to theta = 25.242°	99.8 %	
Absorption correction	Semi-empirical from equivalents	
Max. and min. transmission	0.7452 and 0.7106	
Refinement method	Full-matrix least-squares on F^2	
Data / restraints / parameters	2319 / 240 / 217	
Goodness-of-fit on F^2	1.096	
Final R indices [I>2sigma(I)]	R1 = 0.0350, wR2 = 0.0884	
R indices (all data)	R1 = 0.0386, wR2 = 0.0910	
Extinction coefficient	0.140(7)	
Largest diff. peak and hole	0.206 and -0.197 e.$Å^{-3}$	

5. Supplementary References

1. A. K. Malde *et al.*, An automated force field topology builder (ATB) and repository: version 1.0. *J. Chem. Theory Comput.* **7**, 4026-4037 (2011).
2. N. Schmid *et al.*, Definition and testing of the GROMOS force-field versions 54A7 and 54B7. *Eur. Biophys. J.* **40**, 843-856 (2011).
3. J. J. P. Stewart, MOPAC: A semiempirical molecular orbital program. *J. Comput.-Aided Mol. Des.* **4**, 1-103 (1990).
4. B. Hess, C. Kutzner, D. van der Spoel, E. Lindahl, GROMACS 4: algorithms for highly efficient, load-balanced, and scalable molecular simulation. *J. Chem. Theory Comput.* **4**, 435-447 (2008).
5. M. P. Allen, D. J. Tildesley, *Computer Simulation of Liquids*. (Clarendon Press, 1989).
6. U. Essmann *et al.*, A smooth particle mesh Ewald method. *The Journal of Chemical Physics* **103**, 8577-8593 (1995).
7. B. Hess, H. Bekker, H. J. C. Berendsen, J. G. E. M. Fraaije, LINCS: A linear constraint solver for molecular simulations. *J. Comput. Chem.* **18**, 1463-1472 (1997).
8. S. Nosé, A unified formulation of the constant temperature molecular dynamics methods. *J. Chem. Phys.* **81**, 511-519 (1984).
9. M. Parrinello, A. Rahman, Polymorphic transitions in single crystals: a new molecular dynamics method. *J. Appl. Phys.* **52**, 7182-7190 (1981).
10. R. W. Hockney, J. W. Eastwood, *Computer Simulation Using Particles*. (CRC Press, Bristol, 2010).
11. W. Humphrey, A. Dalke, K. Schulten, VMD: visual molecular dynamics. *J. Mol. Graphics* **14**, 33-38 (1996).
12. G. M. Torrie, J. P. Valleau, Nonphysical sampling distributions in Monte Carlo free-energy estimation: Umbrella sampling. *J. Comput. Phys.* **23**, 187-199 (1977).
13. S. Kumar, J. M. Rosenberg, D. Bouzida, R. H. Swendsen, P. A. Kollman, THE weighted histogram analysis method for free-energy calculations on biomolecules. I. The method. *J. Comput. Chem.* **13**, 1011-1021 (1992).
14. B. Roux, The calculation of the potential of mean force using computer simulations. *Comput. Phys. Commun.* **91**, 275-282 (1995).
15. J. S. Hub, B. L. de Groot, D. van der Spoel, g_wham—A Free Weighted Histogram Analysis Implementation Including Robust Error and Autocorrelation Estimates. *J. Chem. Theory Comput.* **6**, 3713-3720 (2010).
16. Bruker (2014). APEX2. Bruker AXS, Inc., Madison, Wisconsin, USA.
17. Bruker (2014). SADABS. Bruker AXS, Inc., Madison, Wisconsin, USA.
18. Sheldrick,G.M. (2015). Acta Cryst. A71, 3-8.
